# Gene–obesogenic environment interactions in the UK Biobank study

**DOI:** 10.1093/ije/dyw337

**Published:** 2017-01-10

**Authors:** Jessica Tyrrell, Andrew R Wood, Ryan M Ames, Hanieh Yaghootkar, Robin N Beaumont, Samuel E Jones, Marcus A Tuke, Katherine S Ruth, Rachel M Freathy, George Davey Smith, Stéphane Joost, Idris Guessous, Anna Murray, David P Strachan, Zoltán Kutalik, Michael N Weedon, Timothy M Frayling

**Affiliations:** 1 Genetics of Complex Traits, University of Exeter Medical School, University of Exeter, UK,; 2 European Centre for Environment and Human Health, University of Exeter Medical School, The Knowledge Spa, Truro, TR1 3HD, UK,; 3 Wellcome Trust Centre for Biomedical Modelling and Analysis, University of Exeter, RILD Level 3, Exeter, EX2 5DW, UK,; 4 Medical Research Council Integrative Epidemiology Unit at the University of Bristol, Oakfield House, Oakfield Grove, Bristol, BS8 2BN, UK,; 5 Laboratory of Geographical Information Systems (LASIG), School of Architecture, Civil and Environmental Engineering (ENAC), Ecole Polytechnique Fédérale de Lausanne (EPFL), Lausanne, Switzerland,; 6 Unit of Population Epidemiology, Division of Primary Care Medicine, Department of Community Medicine, Primary Care and Emergency Medicine, Geneva University Hospitals and University of Geneva, Geneva, Switzerland,; 7 Department of Ambulatory care and Community medicine, University of Lausanne, Lausanne, Switzerland,; 8 Department of Epidemiology, Emory University, Atlanta, GA, USA,; 9 Population Health Research Institute, St George’s, University of London, Cranmer Terrace, London, SW17 0RE, UK,; 10 Institute of Social and Preventive Medicine (IUMSP), Lausanne University Hospital (CHUV), Lausanne, Switzerland and; 11 Swiss Institute of Bioinformatics, Lausanne, Switzerland

**Keywords:** body mass index, gene–environment, obesogenic environment, social deprivation, UK Biobank

## Abstract

**Background:** Previous studies have suggested that modern obesogenic environments accentuate the genetic risk of obesity. However, these studies have proven controversial as to which, if any, measures of the environment accentuate genetic susceptibility to high body mass index (BMI).

**Methods:** We used up to 120 000 adults from the UK Biobank study to test the hypothesis that high-risk obesogenic environments and behaviours accentuate genetic susceptibility to obesity. We used BMI as the outcome and a 69-variant genetic risk score (GRS) for obesity and 12 measures of the obesogenic environment as exposures. These measures included Townsend deprivation index (TDI) as a measure of socio-economic position, TV watching, a ‘Westernized’ diet and physical activity. We performed several negative control tests, including randomly selecting groups of different average BMIs, using a simulated environment and including sun-protection use as an environment.

**Results:** We found gene–environment interactions with TDI (Pinteraction = 3 × 10^–10^), self-reported TV watching (Pinteraction = 7 × 10^–5^) and self-reported physical activity (Pinteraction = 5 × 10^–6^). Within the group of 50% living in the most relatively deprived situations, carrying 10 additional BMI-raising alleles was associated with approximately 3.8 kg extra weight in someone 1.73 m tall. In contrast, within the group of 50% living in the least deprivation, carrying 10 additional BMI-raising alleles was associated with approximately 2.9 kg extra weight. The interactions were weaker, but present, with the negative controls, including sun-protection use, indicating that residual confounding is likely.

**Conclusions:** Our findings suggest that the obesogenic environment accentuates the risk of obesity in genetically susceptible adults. Of the factors we tested, relative social deprivation best captures the aspects of the obesogenic environment responsible.

## Introduction

The prevalence of obesity is set to dramatically exceed targets set by the World Health Organization and place an increasingly large burden on health services throughout the world.^1^ Whilst environmental influences, including diet and lifestyle, have caused the obesity epidemic,^2^ twin and family studies show that genetic factors influence susceptibility to obesity in today’s environment.^3^^,^^4^ Recent genetic studies have identified many common genetic variants associated with body mass index (BMI)^5^ but the role of genetic susceptibility in different modern-day environments has proven controversial. Different studies have concluded that physical inactivity^6^^,^^7^ and consuming more fried food,^8^ more fizzy drinks^9^ or more protein^10^ accentuates the risk of obesity in those genetically predisposed. These studies have often concluded that their results highlight the need for public health interventions targeted at the specific environmental factors, e.g. ‘highlighting the particular importance of reducing fried food consumption in individuals genetically predisposed to obesity’.^8^ Other studies have not identified interactions, most recently between the *FTO* variant and weight loss.^11^ Previous studies have often had to rely on meta-analysis of data from many heterogeneous studies.^6^^,^^7^^,^^12–14^ Most importantly, unlike main effect Mendelian randomization studies, gene x environment interaction studies are susceptible to confounding.^15^^,^^16^ A recent study, testing only the variant in the *FTO* locus, overcame many of these issues by using a single large, relatively homogeneous study—the UK Biobank—and testing many measures of the environment in the same statistical model.^17^

One objective but broad measure of the obesogenic environment is relative social deprivation. Social deprivation is correlated with obesity in children^18^ and adults,^19^ and studies show that people from more deprived backgrounds make poorer food choices^20^ and tend to be less active.^21^ Whilst people from more socially deprived backgrounds are more overweight on average, few studies have tested the hypothesis that deprivation accentuates genetic susceptibility to obesity. An exception is the recent study using the UK Biobank that nominally suggested that deprivation accentuates the BMI effect of the variant at the *FTO* locus (*P* = 0.035).^17^

The UK Biobank study was designed to improve our understanding of the interaction between genes and the environment in health and disease. It provides a unique opportunity to investigate a range of obesogenic environments and behaviours in a single large, relatively homogeneous study. Here, we hypothesized that genetic susceptibility to high BMI interacts with aspects of the obesogenic environment and obesogenic behaviours to accentuate the risk of obesity.

## Materials and methods

### UK Biobank participants

The UK Biobank recruited over 500 000 adults aged 37–73 years in 2006–10 from across the UK. Participants provided samples and a range of information via questionnaires, interviews and measurements.^22^ We used up to 119 733 adults of White British descent with genetic data, BMI and at least one obesogenic variable available. We did not include other ethnic groups, because individually they were underpowered to detect previously reported effects. British descent was defined as individuals who both self-identified as White British and were confirmed as ancestrally Caucasian using principal components analyses (PCA) of genome-wide genetic information. This dataset underwent extensive central quality control (http://biobank.ctsu.ox.ac.uk) including the exclusion of the majority of third-degree or closer relatives from a genetic kinship analysis of 96% of individuals. We performed an additional round of PCA on these 120 286 UK Biobank participants. We selected 95 535 independent single-nucleotide polymorphisms (SNPs) (pairwise *r*^2^^ ^< 0.1) directly genotyped with a minor allele frequency (MAF) ≥ 2.5% and missingness < 1.5% across all UK Biobank participants with genetic data available at the time of this study (*n* = 152 732), and with HWE *P* > 1 × 10^–6^ within the White British participants. Principal components were subsequently generated using FlashPCA^13^ and the first five adjusted for in all analyses.

### Patient involvement

Details of patient and public involvement in the UK Biobank are available online (http://www.ukbiobank.ac.uk/about-biobank-uk/ and https://www.ukbiobank.ac.uk/wp-content/uploads/2011/07/Summary-EGF-consultation.pdf?phpMyAdmin=trmKQlYdjjnQIgJ%2CfAzik Mh E nx6).

### Phenotypes

#### BMI

The UK Biobank measured weight and height in all participants and calculated BMI. BMI was available for 119 883 individuals of White descent with genetic data available. We performed analyses of BMI on both its natural (kg/m^2^) and an inverse normalized scale to account for differences in variances.

BMI, genetic data and at least one obesogenic measure was available for up to 119 733 individuals (Supplementary Table 1, available as Supplementary data at *IJE* online).

#### Obesogenic environment and behaviour variables

The obesogenic environment refers to an environment that promotes gaining weight and that is not conducive to weight loss.^23^ Here we use the term ‘environment’ to refer to any variable that describes a component to obesity that is not genetic variation. Many of these measures are likely to be a complex mixture of environment and behaviour. For example, the number of fizzy drinks a person consumes could be a mix of availability in the environment and satiety.

We selected 12 measures of the obesogenic environment including Townsend deprivation index (TDI) as a measure of socio-economic position, sedentary time, TV watching, physical activity (three measures), Western diet, percentage protein and fat intake, fried-food consumption, fizzy-drink consumption and a composite score of TV watching, sedentary time, physical activity and Westernized diet. As a negative control, we chose a variable with an implausible causal link to BMI: sun-protection use in the summer. These measures were all self-reported at the same time as BMI was measured with the exception of TDI and the accelerometer data used to measure activity in a subset of individuals (*n* = 19 229). Several measures were correlated with each other, with a maximum correlation of *R* = 0.64 between TV watching and sedentary time (Supplementary Table 2, available as Supplementary data at *IJE* online). For presentation purposes, each obesogenic variable was dichotomized to represent high and low exposure either at the median or a specific cut-off as close to the median as possible. For testing of interactions, we used continuous measures of the environment because using thresholds to select groups of individuals can inflate gene-BMI effect estimates if the variance of the environmental measure is lower in the selected group than the comparison group.

The 12 measures of the obesogenic environment are described below. All self-report measures were associated with factors such as sex, measures of socio-economic position (TDI) and type 2 diabetes in the expected directions, (Supplementary Table 3, available as Supplementary data at *IJE* online).

#### TDI

The TDI is a composite measure of deprivation based on unemployment, non-car ownership, non-home ownership and household overcrowding; a negative value represents high socio-economic position.^24^ TDI was calculated prior to joining the UK Biobank and was based on the preceding national census data, with each participant assigned a score corresponding to the postcode of their home dwelling.

The TDI variable was skewed (Supplementary Figure 1, available as Supplementary data at *IJE* online) and therefore we single inverse normalized this variable for use in sensitivity analyses.

#### Job class

On finding an interaction with TDI, we tested more specific variables related to TDI including job class and number of years in education. The UK Biobank asked people to select their current or most recent job. This was classified into one of the following strata: elementary occupations, process plant and machine operatives, sales and customer service occupations, leisure and other personal service occupations, personal service occupations, skilled trades, admin and secretarial roles, business and public sector associate professionals, associate professionals, professional occupations, and managers and senior officials. Data were available for 76 374 individuals.

#### Years in education

A variable based on the standardized 1997 International Standard Classification of Education (ISCED) of the United Nations Educational, Scientific and Cultural Organisation was created in the UK Biobank, using previously published guidelines.^25^ Data were available for 118 775 individuals.

#### Replication with TDI: CoLaus Study

The CoLaus Study^26^ is a population-based study including over 6500 participants from Lausanne (Switzerland). This study included inhabitants aged 35–75 years at baseline (2003–06) and they were followed up between 2009 and 2012 (mean follow-up 5.5 years). Within this cohort, TDI was available for 5237 individuals with BMI and BMI genetic variants available. The use of TDI in Lausanne may capture socio-economic position in a different way to the UK Biobank, because e.g. not owning a car is not necessarily correlated with precarity. The CoLaus Study complied with Declaration of Helsinki and was approved by the local Institutional Ethics Committee.

#### Replication with job class: 1958 Birth Cohort

The 1958 Birth Cohort^27^ has followed persons born in England, Scotland and Wales during one week in 1958 from birth into middle age. Within this cohort, 6171 individuals had information on social class based on their own current or most recent occupation (at age 42), BMI (measured at age 44–45) and genetic data.

#### Dietary information

All participants completed a generic diet questionnaire during recruitment and a subset of 46 526 individuals completed up to five 24-h food frequency questionnaires (FFQ). The FFQ focused on the consumption of approximately 200 commonly consumed food and drinks (http://biobank.ctsu.ox.ac.uk/crystal/refer.cgi?i =118240). For each participant completing the food frequency questionnaire, nutrient intakes were estimated by multiplying the quantity consumed by the nutrient composition of the food or beverage, as taken from the UK food composition database.^28^ The 46 526 participants with genetic data completing at least one standard (i.e. normal diet) FFQ were included in this study. Where participants had completed more than one FFQ for a standard day’s diet, an average was calculated for the food group of interest.

#### Fizzy-drink consumption

Fizzy-drink consumption was determined from the FFQ and represented number of glasses of fizzy drink consumed on an average day. This was dichotomized at the median, resulting in two groups: low risk (no fizzy drinks daily, *n* = 40 107) and high risk (at least one fizzy drink a day, *n* = 6419). No data on type of fizzy drink were available.

#### Fried-food intake

Fried-food intake was determined from the FFQ and combined the reported intake of fried chicken and fried potato.

#### Percentage fat

Fat (in grams) consumed was taken from the UK Biobank-derived nutrients information in the FFQ. The variable was then divided by total energy intake (in kJ).

#### Percentage protein

Protein (in grams) consumed was taken from the UK Biobank-derived nutrients information in the FFQ. The variable was then divided by total energy intake (in kJ).

#### Calorie-dense ‘Western’ diet

The generic diet questionnaire was used to calculate the average consumption of fruit, vegetables, fish (oily and non-oily), meat (processed, poultry, beef, lamb and pork), cheese, milk, bread, cereal, tea, coffee and water. To condense this information, we performed a principal component factor analysis. Seven eigenvalues were greater than 1, factor 1 was considered to represent a calorie-dense ‘Western’ diet (high intake of prepared meals, processed meats, crisps, etc.) and factor 2 represented a prudent diet (high intake of vegetables, fruit and fish). This information was available for 94 040 individuals of White origin with genetic data available.

### Physical activity

#### International Physical Activity Questionnaire

The UK Biobank asked a range of questions about physical activity questions to all participants. We derived the total metabolic equivalent of task (MET) minutes of exercise per week [based on the International Physical Activity Questionnaire (IPAQ)]. This is calculated using the number of days and minutes per day spent walking, performing moderate or vigorous activity and the speed of walking variable. Individuals reporting more than 16 h of walking and/or moderate and/or vigorous activity a day were excluded (*n* = 1589) on the grounds that these values were likely to be an error or misreporting. All individuals reporting more than 3 h per day of walking, moderate or vigorous activity were re-coded to 3 h as per IPAQ guidelines.^29^

The MET is a physiological measure expressing the energy cost (or calories) of physical activities. The numbers of minutes per week for each level of exercise intensity (walking, moderate and vigorous) are multiplied by specific MET values.^30^ MET values used for the short IPAQ are 2.5 for slow walking, 3.3 for moderate walking and 5 for fast walking, 4 for moderate exercise and 8 for vigorous exercise. Total MET minutes are calculated by summing MET minutes per week for walking, moderate and vigorous exercise. The short form of IPAQ is validated^30^^,^^31^ and utilized in many studies into physical activity.^32^

#### Sedentary behaviour

The UK Biobank asked all participants about the hours per day they spent (i) driving, (ii) using a computer and (iii) watching television. These three variables were summed to provide the hours per day that participants spent sat down. Values greater than 24 h per day were excluded. Those reporting over 16 h were re-coded to 16 h. Sedentary time was available for 119 688 individuals with genetic data available. We dichotomized individuals into those who spent less than 5 h a day sedentary (*n* = 63 631) and those who spent 5 or more hours a day sedentary (*n* = 56 655).

#### TV watching

Participants in the UK Biobank were asked to report how many hours they spent watching TV in a typical day. We dichotomized individuals into those watching 4 or more hours of TV per day (*n* = 37 029) and those watching 3 h or less (*n* = 82 392). This was based on the median value (3 h) but, due to lots of tied values, this resulted in imbalanced groups.

#### Vigorous activity

The minutes of vigorous activity per week were calculated and, for display purposes, a dichotomous variable was also derived denoting participants who performed more than 1 h of vigorous activity per week or not. Of the available individuals, 35 242 reported more than 1 h of vigorous activity per week, whilst 74 128 did not. This was the most balanced way of dichotomizing this variable because only 21 676 individuals reported more than 2 h.

#### Measured physical activity with accelerometer data

Daily accelerometer data were available for 19 229 individuals of White British origin with genetic data available for a period of 6 d. A variable was derived from these data representing the mean levels of moderate physical activity per day for each individual.

#### Composite score of the obesogenic environment and behaviour

Physical activity (as measured by IPAQ), sedentary time, TV watching and Westernized diet were available in 86 549 individuals with BMI genetic variants available. We did not use other variables, as they were only available in smaller numbers. The obesogenic variables were combined using a principle components factor analysis in STATA. Only one factor had an eigenvalue of greater than 1 and this was used as a composite score of the obesogenic environment.

### Negative control ‘environments’

We performed three negative control experiments.

#### Self-reported sun-protection use

First, we used sun-protection use as a negative control variable to assess residual confounding. UK Biobank participants were asked ‘Do you wear sun protection (e.g. sunscreen lotion, hat) when you spend time outdoors in the summer?’ with the options: Never, Sometimes, Most of the time, Always, Don’t go out in the sun, Don’t know and Prefer not to answer. The variable was correlated with TDI and BMI but is implausible as a mechanism (see the Discussion section for why vitamin D exposure is unlikely to be a mechanism in this context) (Supplementary Table 3, available as Supplementary data at *IJE* online).

#### Randomly selecting groups of individuals to be of different average BMI

Second, we used a meta-heuristic sampling approach to randomly select two groups of individuals with BMI distributions identical to the high and low groups for observed obesogenic environment measures. For example, this method was used to select 59 712 individuals with a mean BMI of 27.86 and a standard deviation of 5.12 representing the 50% of individuals in the lowest socio-economic position and a group of 59 754 individuals with a mean BMI of 27.19 and a standard deviation of 4.47 representing the 50% of individuals in the highest socio-economic position*.* There was no overlap between individuals selected for the two groups. Meta-heuristic sampling was repeated 100 times and the interaction *P*-values were calculated each time. Here we report the results from the median analysis based on the interaction *P*-value. We repeated this process 100 times to match average BMIs to those for five dichotomized measures of the environment: four that interacted (at *P* < 0.05): the composite score, self-report physical activity, socio-economic position (TDI) and TV watching; and one that did not interact (at *P* > 0.05) but where BMI differences were substantial: fizzy-drink consumption.

#### BMI GRS interactions with dummy ‘environments’

Third, we created dummy continuous variables as random ‘environments’. The new variables were created in STATA by regressing the obesogenic variables on BMI, the BMI GRS and a range of covariates (age, age^2^, sex) and taking the fitted values and the residuals. The fitted value from the regression was then added to random permutations of the residuals (*n* = 10 000) to produce 10 000 simulated variables that associate with BMI in a similar way to the real obesogenic variable, but are only minimally associated with the real variable itself. This ensures that the simulated variable has the same conditional expectations and same residual distributions as the five real variables (physical activity, TDI, TV watching, the composite score and fizzy-drink consumption). Further information on this method is provided in the Supplementary data (available as Supplementary data at *IJE* online). The interaction model was run for all 10 000 simulations. Here we report the results from the median simulation (based on the interaction *P-*values).

### Selection of genetic variants associated with BMI and GRS

We selected 69 of 76 common genetic variants that were associated with BMI at genome-wide significance in the GIANT consortium in studies of up to 339 224 individuals (Supplementary Table 4, available as Supplementary data at *IJE* online).^5^ We used these variants to create a GRS to represent genetic susceptibility to high BMI—we were not testing specific variants for interaction, but instead how genetic susceptibility overall may be influenced by environmental and behavioural exposures. We used genotypes imputed by UK Biobank. We limited the BMI SNPs to those that were associated with BMI in the analysis of all European ancestry individuals. Variants were excluded if known to be classified as a secondary signal within a locus. Three variants were excluded from the score due to potential pleiotropy [rs11030104 (*BDNF* reward phenotypes), rs13107325 (*SLC39A8* lipids, blood pressure), rs3888190 (*SH2B1* multiple traits)], three SNPs not in Hardy Weinberg Equilibrium (*P* < 1 × 10^–6^; rs17001654, rs2075650, rs9925964) or the SNP was unavailable (rs2033529).

The imputed dosages for each SNP were re-coded to represent the number of BMI-increasing alleles for that particular SNP. A BMI genetic risk score (GRS) was created using the SNPs. Each allele associated with high BMI was weighted by its relative effect size (β-coefficient) obtained from the previously reported BMI meta-analysis data.^5^ A weighted score was created [Equation (1)] in which β is the β-coefficient representing the association between each SNP and BMI:

(1) $$Weighted\;score=\beta_{\text{1}}\times SNP_{\text{1}}+\beta_{\text{2}}\times SNP_{\text{2}}+\ldots \beta_{n}\times SNP_{n}.$$

The weighted score was rescaled to reflect the number of BMI-increasing alleles [Equation (2)]:

(2) $$Weighted\;GRS=\;\frac{weighted\;\;score\;\;x\;\;number\;\;of\;\;available\;\;SNPs}{sum\;\;of\;\;the\;\;\beta \;\;coefficients\;\;of\;\;available\;\;SNPs}.$$

### Statistical analysis

The mean and standard deviation of BMI were calculated in each of the pairs of obesogenic exposures.

For each of the measures of the obesogenic environment, we calculated the association between the 69 SNP BMI GRS and BMI in the high-risk and low-risk environments using linear regression models. BMI was adjusted for age, sex, five ancestry principal components and assessment centre location. We additionally adjusted the full model for genotyping platform (two were used).

Interactions between the genetic variables and the obesogenic environment variables on BMI were tested by including the respective interaction terms in the models [e.g. interaction term = GRS × physical activity (continuous)]. Continuous measures were used to limit spurious results from the gene x environment interactions (Supplementary Methods, available as Supplementary data at *IJE* online).

We performed the analyses in two ways. First, we analysed the data with BMI on its natural scale (kg/m^2^) (residualized for age, sex, centre location and five ancestry principal components). Second, we inverse normalized the data so that BMI, in all 20 strata, had a mean BMI of 0 and a SD of 1. This analysis allowed us to account for the differences in BMI variation observed in high- and low-risk strata. We present primary results from the inverse normalized data. To further assess the extent to which differences in BMI variation could influence our results, we tested for heteroscedasticity using the Breusch-Pagan test as implemented with the estat hettest in STATA.^33^ Standard regression analysis can produce biased standard errors if heteroscedasticity is present.^34^ If heteroscedasticity was present, we used robust standard errors, using the vce(robust) option in STATA, which relaxes the assumption that errors are both independent and identically distributed and are therefore more robust.

For the TDI analyses, we also repeated the analysis adjusting for other measures of the environment previously associated with interactions, including self-reported physical activity, TV watching and diet^7^^,^^9^^,^^10^^,^^35^ and corrected for interaction terms with other environmental measures.

Finally, we investigated each of the 69 SNPs individually. Interactions between each SNP and the TDI on BMI were tested by including the respective interaction terms in the models [e.g. interaction term = SNP × TDI (continuous)].

Identical analyses were performed in the CoLaus Study and the 1958 Birth Cohort.

### Testing for potential reverse causality

Genetic variants could influence BMI through primary effects on physical activity or diet-related variables, especially when BMI is measured at the same time as the exposure. For example, alleles that reduce activity could increase BMI and result in estimates of self-reported activity biased towards higher activity. This direction of causality could result in alleles associated with higher BMI being associated with stronger effects on BMI in people reporting more activity. To attempt to test for this possibility, we looked for evidence that BMI-associated variants had primary effects on levels of activity and measures of diet. None of the BMI-associated variants had effects on activity that were disproportionately larger than their BMI effects (Supplementary Methods and Supplementary Figure 2, available as Supplementary data at *IJE* online). The BMI GRS was associated with some of the obesogenic measures of the environment (3 of 12 below the threshold of 0.004; Supplementary Table 5, available as Supplementary data at *IJE* online).

## Results

### Measures of the obesogenic environment and behaviour are associated with BMI and variance in BMI in the UK Biobank study

We used 12 measures of the obesogenic environment and behaviour that were associated with BMI in the UK Biobank in the expected directions (Table 1). All self-reported measures were associated with sex, measures of socio-economic status and type 2 diabetes in the expected directions, suggesting that over-reporting of healthy and underreporting of unhealthy behaviour had not completely biased the associations with self-reported measures (Supplementary Table 3, available as Supplementary data at *IJE* online). In each case, the group of people in the higher-risk environment had a larger mean BMI but also a larger variation in BMI, as measured by the standard deviation, compared with people in the lower risk environment (Table 1 and Supplementary Figure 3, available as Supplementary data at *IJE* online). For example, the 50% least (self-reporting) physically active people (*n* = 54 569) had an average BMI of 27.9 kg/m^2^, and 95% had a BMI between 21.3 and 37.3 kg/m^2^ (a range of 16) whereas the 50% most physically active people (*n* = 54 573) had an average BMI of 26.9 kg/m^2^, and 95% had a BMI between 21.9 and 34.7 kg/m^2^ (a range of 12.8).

**Table 1.** Comparison of the high- and low-risk categories for the 10 obesogenic environmental/behavioural measures, the composite score and the negative control (sun protection)

Environmental factor	Obesogenic category	*N*	Male, *N* (%)	Mean BMI	SD BMI	Effect size (95% CI) representing change in BMI (kg/m^2^) for people in the high-risk group compared with the low-risk group^a^	*P*
Fizzy drink	None daily	39 975	18 327 (45.9)	26.93	4.62	Reference	
	≥1 glass daily	6393	3537 (55.3)	27.69	4.91	0.71 (0.58, 0.83)	<1E-15
Fried-food intake	None daily	31 821	14 485 (45.5)	26.96	4.66	Reference	
	≥1 meal daily	14 547	7379 (50.7)	27.20	4.68	0.20 (0.10, 0.29)	0.00002
Percentage fat^b^	Low risk	23 194	11 080 (47.8)	26.91	4.46	Reference	
	High risk	23 174	10 784 (46.5)	27.16	4.86	0.28 (0.19, 0.36)	1E-10
Percentage protein^b^	Low risk	23 188	12 137 (52.3)	26.70	4.54	Reference	
	High risk	23 180	9727 (42.0)	27.37	4.77	0.77 (0.68, 0.85)	<1E-15
Western diet^b^	Low risk	47 027	19 783 (42.1)	27.06	4.71	Reference
	High risk	47 013	24 853 (52.9)	28.00	4.79	0.86 (0.80, 0.92)	<1E-15
IPAQ	>1845 MET min/week	54 573	27 217 (49.9)	26.86	4.31	Reference	
	≤1845 MET min/week	54 569	25 111 (46.0)	27.93	4.99	1.11 (1.06, 1.17)	<1E-15
Sedentary time	<5 h daily	63 343	25 281 (39.9)	26.61	4.47	Reference
	≥5 h daily	56 345	31 387 (55.7)	28.56	4.99	1.84 (1.78, 1.89)	<1E-15
TV	<4 h daily	82 022	38 866 (47.4)	26.98	4.54	Reference
	≥4 h daily	36 814	17 496 (47.5)	28.70	5.16	1.69 (1.63, 1.75)	<1E-15
Vigorous activity	>1 h weekly	35 242	18 672 (53.0)	26.81	4.24	Reference
	≤1 h weekly	74 128	33 760 (45.5)	27.69	4.88	0.92 (0.86, 0.98)	<1E-15
Measured physical activity^b^	Low risk	9632	4038 (41.9)	25.79	3.92	Reference	
	High risk	9636	4777 (49.6)	27.79	4.92	1.97 (1.84, 2.09)	<1E-15
TDI (natural scale)	High SEP TDI < –2.294	59 872	28 383 (47.4)	27.20	4.47	Reference	
	Low SEP TDI > –2.294	59 861	28 306 (47.3)	27.87	5.13	0.69 (0.64, 0.75)	<1E-15
Composite score^b^	Low risk	43 275	19 768 (45.7)	26.33	4.13	Reference	
	High risk	43 274	21 933 (50.7)	28.46	4.87	2.08 (2.02, 2.14)	<1E-15
Sun-protection use	Usually or always use	68 507	25 641 (37.4)	27.32	4.75	Reference	
	Never or sometimes use	50 561	30 743 (60.8)	27.81	4.89	0.31 (0.25, 0.37)	<1E-15

^a^Adjusted for age, sex and ancestry principal components.

^b^High and low risk taken from median values.

### Genetic variants are associated with BMI in the UK Biobank study

The BMI GRS, consisting of 69 known BMI-associated variants, was associated with higher BMI and explained 1.5% of the variation in BMI—a figure consistent with previous studies.^5^

### Measures of high-risk obesogenic environments and behaviours are associated with an accentuated risk of high BMI in genetically susceptible individuals

**Figure 1.**
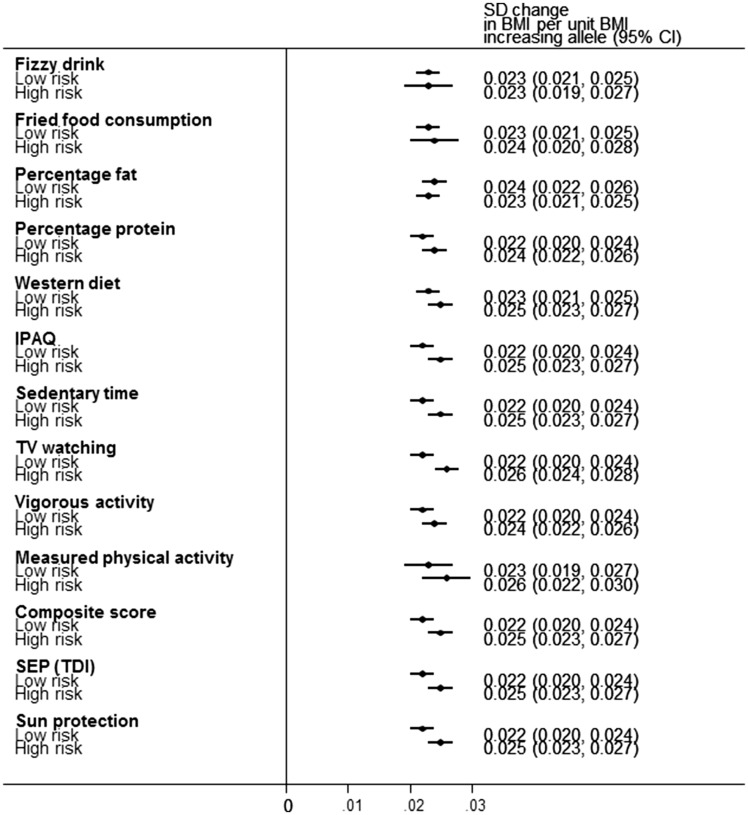
Forest plot demonstrating the change in BMI per-allele increase in BMI genetic risk score (GRS) for the 12 different obesogenic environments and the negative control on a standardized inverse normalized scale. BMI was corrected for age, sex, ancestry principal components and assessment centre location prior to calculating residuals. The analyses were further adjusted for genotype platform.

**Figure 2.**
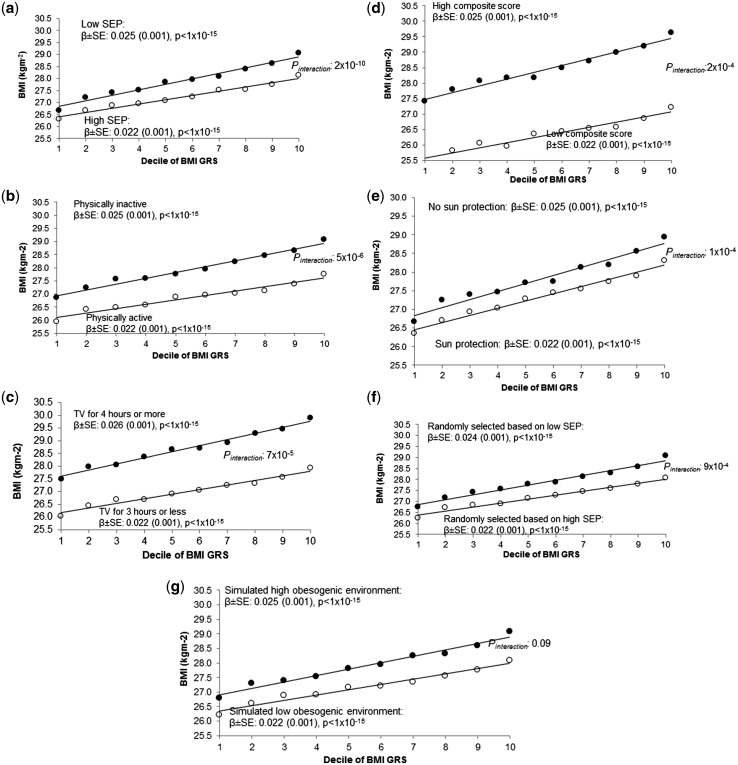
Association between the BMI GRS (by decile) and BMI in (a) the most socially deprived (black circles) and least socially deprived (white circles); (b) high and low self-reported physical activity, (c) high and low TV watching and (d) high and low composite score, (e) high and low use of sun protection in the summer, (f) individuals randomly selected to be of high BMI (black circles) and individuals randomly selected to be of low BMI (white circles) and (g) individuals in the high obesogenic simulated environment (black circles) and individuals in the low obesogenic simulated environment (white circles). Note that, for the simulated environment, we used the median BMI GRS BMI association after 1000 simulations. For (f), it was not possible to use a continuous measure in the calculation of the interaction term. This figure is based on a similar way of showing interaction data with a BMI GRS from ^12^. SEP, socioeconomic position.

We observed interactions between measures of the obesogenic environment and genetic susceptibility to high BMI in the following scenarios (Table 2, Figures 1 and 2, and Supplementary Figure 4, available as Supplementary data at *IJE* online).

**Table 2.** Differences in BMI by BMI GRS decile (kg/m^2^) and by allele (inverse normalized scale) for the obesogenic environmental/behavioural measures, the composite score and the negative control (sun protection)

Trait	Obesogenic category	*N*	BMI difference in 10% lowest genetic risk	BMI difference in 10% highest genetic risk	Per-allele beta	SE	*P* association	*P* interaction^a^	*P* interaction robust^b^
Fizzy drink	None daily	39 975	+0.93 kg/m^2^	+0.79 kg/m^2^	0.023	0.001	<1 × 10^–15^	0.86	0.86
	≥1 glass daily	6393			0.023	0.002	<1 × 10^–15^		
Fried-food consumption	None daily	31 821	+0.35 kg/m^2^	+0.52 kg/m^2^	0.023	0.001	<1 × 10^–15^	0.94	0.94
	≥1 meal daily	14 547			0.024	0.002	<1 × 10^–15^		
Percentage fat^c^	Low risk	23 194	+1.91 kg/m^2^	+2.10 kg/m^2^	0.024	0.001	<1 × 10^–15^	0.58	0.59
	High risk	23 174			0.023	0.001	<1 × 10^–15^		
Percentage protein^c^	Low risk	23 188	+1.90 kg/m^2^	+2.10 kg/m^2^	0.022	0.001	<1 × 10^–15^	0.78	0.79
	High risk	23 180			0.024	0.001	<1 × 10^–15^		
Western diet^c^	Low risk	47 027	+0.76 kg/m^2^	+1.02 kg/m^2^	0.023	0.001	<1 × 10^–15^	0.05	0.07
	High risk	47 013			0.025	0.001	<1 × 10^–15^		
IPAQ	>1845 MET min/week	54 573	+0.92 kg/m^2^	+1.32 kg/m^2^	0.022	0.001	<1 × 10^–15^	**2 × 10^–^^6^**	**5 × 10^–^^6^**
	≤1845 MET min/week	54 569			0.025	0.001	<1 × 10^–15^		
Sedentary time	<5 h daily	63 343	+1.73 kg/m^2^	+2.13 kg/m^2^	0.022	0.001	<1 × 10^–15^	**0.023**	**0.030**
	≤5 h daily	56 345			0.025	0.001	<1 × 10^–15^		
TV watching	<4 h daily	82 022	+1.46 kg/m^2^	+1.97 kg/m^2^	0.022	0.001	<1 × 10^–15^	**1 × 10^–^^5^**	**7 × 10^–^^5^**
	≤4 h daily	36 814			0.026	0.001	<1 × 10^–15^		
Vigorous activity	>1 h weekly	35 242	+0.72 kg/m^2^	+1.05 kg/m^2^	0.022	0.001	<1 × 10^–15^	**0.008**	**0.013**
	≤1 h weekly	74 128			0.024	0.001	<1 × 10^–15^		
Measured physical activity^a^	Low risk	9632	+1.63 kg/m^2^	+2.53 kg/m^2^	0.023	0.002	<1 × 10^–15^	0.10	0.11
	High risk	9636			0.026	0.002	< 1 × 10^–15^		
TDI (natural scale)	High SEP TDI ≤ –2.294	59 872	+ 0.35 kg/m^2^	+ 0.92 kg/m^2^	0.022	0.001	<1 × 10^–15^	**6 × 10^–^^12^**	**2 × 10^–^^10^**
	Low SEP TDI > –2.294	59 861			0.025	0.001	<1 × 10^–15^		
Composite score^c^	Low risk	43 275			0.022	0.001	<1 × 10^–15^	**1 × 10^–^^4^**	**2 × 10^–^^4^**
	High risk	43 274			0.025	0.001	<1 × 10^–15^		
Sun-protection use	Usually or always use	68 507	+0.32 kg/m^2^	+0.63 kg/m^2^	0.022	0.001	<1 × 10^–15^	**1 × 10^–^^4^**	**1 × 10^–^^4^**
	Never or sometimes use	50 561			0.025	0.001	<1 × 10^–15^		

BMI adjusted for age, sex, ancestral principal components and assessment centre location and then inverse normalized. Models additionally adjusted for genotyping platform.

^a^Interaction *P*-value.

^b^Interaction *P*-value accounting for heteroscedasticity using robust standard errors.

^c^Data were split on the basis of arbitrary median values.

#### TDI

A higher level of deprivation was associated with an accentuated genetic susceptibility to higher BMI. The effect of the BMI GRS on BMI was larger in the group of 50% living in the most relatively deprived situations {0.025 standard deviations per allele [95% confidence interval (CI): 0.023–0.027]} compared with the group of 50% living in the least deprived situations [0.022 SDs per allele (95% CI: 0.020–0.024)] (Table 2 and Figure 2a). When performing the analysis with TDI on a continuous scale (a more robust analysis than using dichotomized measures), the interaction was strong: *P_interaction_* 2 × 10^–10^. This apparent gene x deprivation interaction meant that, compared with below-average deprivation (in the UK Biobank), above-average deprivation was associated with a 0.92 kg/m^2^ higher BMI in people with the highest genetic risk (top decile) but a 0.35 kg/m^2^ higher BMI in people at least genetic risk (bottom decile) (Table 2 and Figure 2a). Another way of expressing the interaction is that, within the 50% group living in the most deprived situations, carrying 10 additional BMI-raising alleles (weighted by effect size) was associated with 3.8 kg extra weight in someone 1.73 m tall. In contrast, within the 50% group living in the least deprived situations, carrying 10 additional BMI-raising alleles was associated with 2.9 kg extra weight in someone 1.73 m tall. These differences were even stronger when using a cut-off that reflected the UK population average TDI^36^ (Supplementary Table 6, available as Supplementary data at *IJE* online) and were consistent across different age groups (Supplementary Table 7, available as Supplementary data at *IJE* online). We also noted that the interaction effect was not driven by specific BMI-associated variants, but was a feature of general genetic susceptibility to higher BMI, as measured by the 69 SNP BMI risk score (Supplementary Table 8 and Supplementary Figure 5, available as Supplementary data at *IJE* online). Excluding the *FTO* variant did not alter the evidence of interaction.

In the CoLaus Study of 5237 individuals from Switzerland, we did not observe any TDI–BMI GRS interaction, but the effect estimates overlap those in the UK Biobank (Supplementary Table 9, available as Supplementary data at *IJE* online).

#### Lower occupational job class and less time spent in education were not associated with an accentuated genetic susceptibility to higher BMI

To further explore possible reasons for the TDI interaction, we tested job class and time spent in education. In both the UK Biobank and the 1958 Birth Cohort, people with lower job classes had a higher mean and standard deviation for BMI. However, there, we found no interaction between job class and GRS in determining BMI in either study (Supplementary Table 9, available as Supplementary data at *IJE* online). Using the UK Biobank data, there was no interaction between time in education and GRS in influencing BMI (Supplementary Table 9, available as Supplementary data at *IJE* online).

#### Self-reported physical activity

The effect of the BMI GRS on BMI was larger in the 50% of people reporting less physical activity [0.025 standard deviations per allele (0.023–0.027)] compared with the 50% reporting more physical activity [0.022 (0.020–0.024)] (*P_interaction_* 5 × 10^–6^; IPAQ on a continuous scale) (Table 2 and Figure 2b).

In a subsample (*n* = 19 229) of people we used an objective, accelerometer-based measure of physical activity recorded over 6 d. We noted a similar trend with a larger effect of the BMI GRS on BMI in less physically active people [0.026 standard deviations per allele (0.022–0.029)] compared with those doing more physical activity [0.023 (0.019–0.027)], although the evidence of interaction was weak (*P_interaction_* 0.11; Table 2).

#### TV watching

The effect of the BMI GRS on BMI was larger in people watching 4 or more hours of TV per day [0.026 standard deviations per allele (0.024–0.028)] compared with those watching 3 h or less [0.022 (0.021–0.024)] (*P_interaction_* 7 × 10^–5^; using TV watching on a continuous scale) (Table 2 and Figure 2c).

#### Other self-reported measures of the obesogenic environment

We did not find any gene x obesogenic environment interaction when considering sedentary time, vigorous activity, Westernized diet, percentage protein or fat in diet, fried-food or fizzy-drink consumption at Bonferroni-adjusted thresholds (*P* < 0.004; Table 2). In six of these seven measures (exception percentage fat consumption), the trend was towards the high-risk obesogenic environments accentuating the risk of high BMI in genetically susceptible individuals.

#### A composite measure of the obesogenic environment

We next tested a composite score consisting of four self-report variables available in the majority of people: sedentary time, TV watching, physical inactivity and Westernized diet. The 50% of people with a high composite score were on average 2.2 kg/m^2^ BMI units heavier than the 50% with a low composite score. The effect of the BMI GRS on BMI was larger in people with a high composite score [0.025 standard deviations per allele (0.023, 0.027)] compared with those with a low composite score [0.022 (0.021–0.024)] (*P_interaction_* 2 × 10^–4^; composite score on a continuous scale) (Table 2 and Figure 2d).

#### The gene x environment interactions may not be specific to the environments tested: using negative controls

We next hypothesized that the interactions observed may not be specific to the obesogenic environment tested, but a general feature of selecting groups of individuals of higher BMI and comparing them to groups of individuals of lower BMI. For example, previous studies have observed stronger effects of BMI-raising alleles in groups of individuals who are less active, eating more fried food and consuming more sugary drinks.^6^^,^^9^^,^^35^ However, all these groups were more overweight on average than those with the healthier lifestyles and environments, and any interaction observed may have been a feature of higher BMI and the general environment, not the specific environment tested. We therefore performed three additional, negative control analyses to test the specificity of the interactions observed. These tests represented ‘impossible by the proposed mechanism’ negative controls.^37^^,^^38^ These analyses also help to test whether or not statistical artefacts were influencing our results, such as different variances in BMI.

#### Sun-protection use as a negative control

First, we tested sun-protection use as a negative control that has no plausible role in obesity but is associated with deprivation, the measure with the strongest evidence of interaction. Using less sun protection in the summer was associated with higher deprivation and there was an interaction with genetic susceptibility to higher BMI, before (*P_interaction_* 1 × 10^–4^) and after adjustment for TDI (Table 2 and Figure 2e).

#### Individuals randomly selected to be of different BMIs

**Figure 3.**
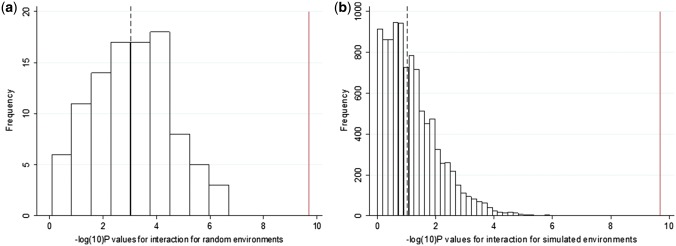
Histograms showing the -log10(*P-*values) for the interactions from (a) the 100 iterations of the individuals selected to be of different BMIs at random and (b) the 10 000 iterations of a simulated environment with a similar association to BMI as TDI. The dashed line represents the median value and the solid line represents the *P*-value obtained from the real interactions with TDI.

Second, we sampled individuals so that they had identical BMI distributions (means and standard deviations) to the high and low TDI groups, but were otherwise randomized to all other variables. We then tested for evidence of interaction using these randomly selected groups. These analyses were repeated 100 times. The associations between the BMI GRS and BMI in these randomly selected individuals were similar to those observed when we selected based on Townsend deprivation index, but none of the 100 iterations showed an interaction *P*-value lower than the real TDI interaction (median *P* = 9 × 10^–4^; Table 3, Figure 2f and Figure 3a). We repeated this analysis by selecting individuals to have similar BMI distributions to those in the high- and low-physical-activity, TV-watching, fizzy-drink-consumption or the high- and low-composite-score groups but who were otherwise randomized to all other variables. We saw some interaction with the BMI GRS having larger effects on BMI in the fatter group compared with thinner group (median of 100 permutations *P* = 0.003, *P* = 0.047 and *P* = 0.028 for those selected to have similar BMIs to the physical activity (IPAQ), TV-watching and composite-score groups, respectively) (Table 3 and Supplementary Figure 6, available as Supplementary data at *IJE* online). No interaction was found for groups based on the high- and low-fizzy-drink groups (a real variable with no evidence of interaction) (Table 3 and Supplementary Figure 6, available as Supplementary data at *IJE* online). We note that these analyses are not completely representative of the real analyses because the interaction term is a binary variable (presence or absence of the individual in the randomly selected groups of higher and lower BMI), not continuous.

**Table 3.** Associations between BMI GRS and BMI (inverse normalized scale) when randomly selecting groups of different BMIs or using a simulated environment. The randomly selected groups and simulated environments were based on the observed BMI distributions in the ‘Trait based on’ column

Simulation	Trait based on	Simulation category	*N*	BMI (SD)	Beta (per allele)	SE	*P* association	*P* interaction^a^	*P* interaction robust^b^
Randomly selected individuals^c^	TDI	Low risk	59 753	27.19	0.022	0.001	<1 × 10^–15^	**8** × **10^–4^**	**9** × **10^–4^**
				(4.47)					
		High risk	59 711	27.86	0.024	0.001	<1 × 10^–15^		
				(5.12)					
Simulated environment	TDI	Low risk	59 741	27.16	0.022	0.001	<1 × 10^–15^	0.09	0.10
				(4.61)					
		High risk	59 740	27.90	0.025	0.001	<1 × 10^–15^		
				(5.01)					
Randomly selected individuals^c^	IPAQ	Low risk	54 573	26.86	0.022	0.001	<1 × 10^–15^	**0.002**	**0.003**
				(4.31)					
		High risk	54 519	27.93	0.024	0.001	<1 × 10^–15^		
				(4.99)					
Simulated environment	IPAQ	Low risk	59 979	26.97	0.022	0.001	<1 × 10^–15^	**0.022**	**0.025**
				(4.48)					
		High risk	59 978	28.11	0.025	0.001	<1 × 10^–15^		
				(5.08)					
Randomly selected individuals^c^	TV watching	Low risk	82 022	26.98	0.023	0.001	<1 × 10^–15^	**0.044**	**0.047**
				(4.54)					
		High risk	36 814	28.70	0.025	0.001	<1 × 10^–15^		
				(5.16)					
Simulated environment	TV watching	Low risk	59 392	26.59	0.023	0.001	<1 × 10^–15^	0.07	0.08
				(4.34)					
		High risk	59 391	28.47	0.024	0.001	<1 × 10^–15^		
				(5.06)					
Randomly selected individuals^c^	Composite score	Low risk	43 275	26.33	0.021	0.001	<1 × 10^–15^	**0.027**	**0.028**
				(4.13)					
		High risk	43 274	28.46	0.023	0.001	<1 × 10^–15^		
				(4.87)					
Simulated environment	Composite score	Low risk	59 844	27.21	0.023	0.001	<1 × 10^–15^	**0.002**	**0.003**
				(4.64)					
		High risk	59 844	27.85	0.024	0.001	<1 × 10^–15^		
				(4.97)					
Randomly selected individuals^c^	Fizzy drink	Low risk	39 975	26.93	0.023	0.001	<1 × 10^–15^	0.47	0.48
				(4.62)					
		High risk	6393	27.69	0.025	0.002	<1 × 10^–15^		
				(4.91)					
Simulated environment	Fizzy drink	Low risk	37 103	26.66	0.024	0.001	<1 × 10^–15^	0.26	0.30
				(4.31)					
		High risk	9275	28.58	0.024	0.001	<1 × 10^–15^		
				(5.64)					

BMI adjusted for age, sex, ancestral principal components and assessment centre location. Models additionally adjusted for genotyping platform.

^a^Interaction *P*-value.

^b^Interaction *P*-value accounting for heteroscedasticity using robust standard errors.

^c^By Meta-heuristic sampling.

#### A dummy environment

Third, we generated a dummy continuous environment associated with BMI but not TDI, physical activity or any of the other measures of the obesogenic environment. We forced this variable to have a similar correlation to BMI as the observed real TDI, physical-activity, TV-watching, the composite-score and the fizzy-drink variables, but that was only very minimally associated with those real measures of the environment (see the ‘Methods’ section). We then tested the hypothesis that the BMI GRS would have stronger effects on BMI in the individuals ‘exposed’ to high levels of this dummy obesogenic environment. We observed some interaction, with the BMI GRS having stronger effects on BMI in the fatter groups (*P* = 0.10, *P* = 0.025, *P* = 0.08 and *P* = 0.003 for the dummy environments correlated with BMI to the same extent as TDI, physical activity, TV watching and the composite score, respectively, based on the median of 10 000 dummy environments tested) (Figure 2g, Figure 3b, Table 3 and Supplementary Figure 7, available as Supplementary data at *IJE* online). No interaction was observed for the dummy environment correlated with BMI to the same extent as fizzy drinks (Table 3 and Supplementary Figure 7, available as Supplementary data at *IJE* online). However, the evidence of interaction with these dummy environments tended to be weaker than that for the real variables. For example, in the 10 000 permutations of a dummy environment, we never observed interactions as strong as that observed with real TDI, providing evidence at *P* < 0.0001 that the TDI effect was capturing a genuine interaction (Figure 3b).

#### Sensitivity analyses

We next performed several sensitivity analyses to further test the interaction of TDI, TV-hours, physical activity and a composite measure of the obesogenic environment with the BMI GRS. We explored a potential source of error—the correlation between the risk factors and the outcomes. In this study, risk factors in the interaction model—measures of the obesogenic environment—were associated with the outcome—BMI. In theory, this problem could have created false positive interactions but a number of sensitivity analyses suggested that this was not the case (Supplementary information and Supplementary Table 10, available as Supplementary data at *IJE* online). We showed that the interactions for each of the four measures (IPAQ, TDI, TV watching and the composite score) were similar when correcting for smoking and the other three measures. We also showed that the interaction with TDI remained strong when correcting for the interaction terms of the other three variables. In contrast, the interaction was attenuated for IPAQ, TV watching and the composite score when including the TDI interaction term (Supplementary Table 11, available as Supplementary data at *IJE* online).

#### Inflated interactions when analysing BMI on the kg/m^2^ scale

When analysed on the natural BMI scale (kg/m^2^), the evidence of interaction was stronger than when using an inverse normalized scale, but likely partly artefactual. The BMI GRS was associated with even larger effects on BMI in high-risk obesogenic environments compared with low-risk environments, and there were apparent interactions (at *P* < 0.05) in seven of the 12 tests (Supplementary Table 12 and Supplementary Figure 8, available as Supplementary data at *IJE* online). This potential artefact occurs because the variance in BMI was higher in individuals in the high-risk environment groups and this heteroscedasticity inflates effect estimates (Supplementary Figure 9, available as Supplementary data at *IJE* online).

## Discussion

In the UK Biobank, we found that aspects of the obesogenic environment accentuate genetic susceptibility to higher BMI. The corollary of this finding, if true, is that exposure to low-risk obesogenic environments partially attenuates the effects of genetic susceptibility to obesity. Of the factors we tested, relatively low socio-economic position, as measured by the TDI, best captured the relevant environmental factors. Our results provide some evidence for public health campaigns aimed at reducing obesity but suggest that measures that target more deprived individuals may have proportionally higher impact. We were not testing for specific gene variant–environment interactions, but instead asking a question of public health relevance—are people at higher risk of obesity due to their genetics more susceptible to the obesogenic environment? We used a BMI GRS as a measure of genetic susceptibility, and the data suggested that no individual variants contributed disproportionately to the evidence of interaction.

The relevant components of higher levels of deprivation that accentuate the genetic risk of obesity are uncertain. When adjusting for measures of self-report physical activity, a more calorie-dense ‘Westernized’ diet and sedentary activity, the evidence of interaction remained strong. This observation, and the interaction with a composite score, suggests that no one aspect of the obesogenic environment we tested can explain the interaction effect with TDI, although a caveat to this argument is that these other measures were self-reported. This conclusion contrasts with those from some previous studies that have suggested (in separate papers) that fried-food and sugary-drink consumption and TV watching specifically interact with BMI genetics.^7–10^^,^^35^ The evidence of interaction remained strong when adjusting for urban vs rural dwelling—an objective measure associated with obesity in the UK Biobank and previously proposed as a contributory factor to the obesogenic environment (through reduced exposure to open spaces, e.g. ^39^).

Our results are consistent with data from twins, where the genetic component to obesity is stronger in young UK children exposed to the modern environment (twins born in the 1990s and measured at the age of 9), compared with measures from twin studies in earlier generations^3^ and that the genetic and environmental components to common traits varies by UK region.^40^

The use of negative controls provided two additional pieces of evidence about the nature of the gene x obesogenic environment interactions. First, when compared with the real data, the evidence of interaction was weaker when using a simulated environment or randomly selecting groups to be of different BMIs. For example, for TDI, we never observed the actual interaction in 10 000 simulations of a dummy environment or 100 iterations of selecting groups of different BMIs. These control experiments mimicked almost perfectly the observed differences in BMI, but still the evidence of interaction was weaker than when using the real obesogenic environments. These results suggest that *something* about the real obesogenic environment, captured by TDI, accentuates genetic risk of obesity.

Second, the use of a control measure implausibly linked to obesity, sun-protection use, helped us establish the possibility that residual confounding has affected the results. The importance of using negative controls in epidemiology to control for this residual confounding has been discussed^37^^,^^38^ and is closely related to the use of one of Hill’s original criteria for causal inference in epidemiology—that of specificity of the exposure–outcome association.^41^ The fact that this negative control showed evidence of interaction, even after adjustment for TDI, suggests that either it is a bad negative control or it is correlated with other obesogenic factors not captured by TDI—residual confounding. We believe that sun-protection use is a good negative control: low vitamin D levels (which would be caused by high use of sun protection) are associated with higher BMI, but there is genetic evidence that this is not a causal relationship^42^ and, even if it were, would have resulted in evidence of interaction in the opposite direction to our observation.

The observation of some evidence of interaction in all our negative control experiments indicates that genetic variants altering BMI may have larger effects in any group of individuals of higher BMI compared with those with lower BMI. Our results show that the greater the mean and variance of BMI, the greater the apparent effects of genetic variants. These effects may be driven by statistical artefacts that can affect gene x environment interaction studies, and we note that the evidence is sensitive to the scale on which the non-genetic factors are analysed. Further work, including the use of negative controls that are likely associated with unmeasured confounders but are implausible, will help disentangle which aspects of the environment are causally interacting with BMI genetics to accentuate the risk of high BMI.

Our analysis had a number of strengths. The major strength was the availability of a single large study, which was beneficial for two main reasons. First, it provided us with relatively homogenous measures of the environment. Several previous studies were limited to meta-analyses of summary statistics from many studies with heterogeneous measures of the environment.^6^^,^^8–10^ An exception is a recent study that also used the UK Biobank and individual-level data to jointly model multiple exposures and provide evidence that some measures that we did not test, including frequency of alcohol consumption and adding salt to food, remain interacting when adjusting for TDI.^17^ Second, it allowed us to test the robustness and specificity of our results by using a composite measure of the environment, randomly selecting individuals and testing interactions using a dummy, simulated environment. A third advantage is that we used an objective measure of the environment: TDI, which provides a cleaner interpretation of results compared with those from previous studies that have had to rely on subjective measures such as self-reported diet and physical activity. These subjective measures are often complex mixtures of environment and behaviour and may be subject to reporting biases. The fourth advantage of our study is that we used a negative control variable—sun-protection use—which helps control for residual confounding. Finally, we performed extensive analyses to account for potential statistical artefacts that can plague gene x environment interaction studies. For example, we have accounted for the effects of heteroscedasticity—a statistical term that describes unequal variance in data. Groups of overweight individuals have a wider variance in BMI than groups of thinner individuals and these differences in BMI can create false positive evidence of interaction. Previous studies have not necessarily accounted for these ‘scale’ effects and are likely to have overestimated the effects of any interactions.

The major limitation of our study, as with most previous studies, is that the majority of the obesogenic variables were based on self-reported measures, and that these self-reports were made at the same time as BMI was measured. A more objective measure of physical activity demonstrated similar results to the self-reported physical activity, but accelerometer-based measures of activity were only available in one-fifth of the dataset. Other limitations of our study include (i) the possibility of reverse causality—genetic variants that predispose to higher BMI may in turn lead to a stronger association with BMI if they make people less active (Supplementary Table 9, available as Supplementary data at *IJE* online); (ii) subtle effects—from Figure 3, we can see that the correlation between BMI genetics and BMI is only slightly larger in the high-risk compared with low-risk environment groups. However, the differences are still such that carrying an additional 10 BMI-raising alleles can increase weight by up to 3.6 kg in a high-risk environment compared with 2.8 kg in a low-risk environment (for a person of average height); (iii) the use of cross-sectional data, with self-reported measures of the obesogenic environment made at the same time as BMI was measured—bias may be introduced by individuals with higher BMIs trying to lose weight through diet and exercise; (iv) missing data—not all participants responded to diet and physical activity questions which may introduce further bias into the study; individuals not reporting were more likely to be older, female and with higher BMI; and (v) the measures of the obesogenic environment were correlated with each other and therefore the tests were not independent. For example, TV watching and sedentary time were the most correlated measures (*r* = 0.64). We also cannot rule out collider bias^43^ affecting the results because individuals participating in the UK Biobank study are biased towards those from higher socio-economic positions and with lower BMIs.

Our results provide an advance for gene x environment interaction studies. We highlight many of the statistical and methodological issues that can make interpretation of GxE results very difficult. One aspect that we can be very confident about, and that contrasts with the conclusions from previous studies, is that there is no evidence that one particular aspect of the environment or behaviour, if altered, would have a preferential benefit over others. It is premature to use genetic interaction studies to suggest that public health measures should be targeted specifically at fried-food reduction, fizzy-drink consumption or diet in those genetically predisposed to obesity.^8^^,^^9^ However, our data suggest that *something* about the obesogenic environment accentuates the genetic susceptibility to obesity and that, of the factors we tested, socio-economic position best captures the relevant factors.

## Supplementary Data

Supplementary data are available at *IJE* online.

## Supplementary Material

Supplementary Data
