# Supplementary material for: Gene–obesogenic environment interactions in the UK Biobank study
Source: Int J Epidemiol. 2017 Jan 10;46(2):559–75. doi: 10.1093/ije/dyw337 (PMC5837271; doi:10.1093/ije/dyw337)
Supplement: Supplementary Data [file dyw337_supp.docx]

Supplementary Information

**Generating the simulated variables for TDI, physical activity, TV watching and the composite score**

In our analysis of gene x environment interactions for TDI, physical activity, TV watching and the composite score the basic starting equation is as follows:

$\left( \boldsymbol{y} | \boldsymbol{C,g,e} \right)\boldsymbol{=C.c+\alpha g+\beta e+\varepsilon}$ (1)

where **y** is BMI, **e** is the environmental variable (in this case TDI, TV watching, physical activity or the composite score), **g** is the BMI genetic risk score and **C** represents important covariates.

Since **g** is a genetic risk score for the trait **y**, *α* is non-zero but *β* can be zero. When statistical interaction in tested, the model is changed to

$\left( \boldsymbol{y} | \boldsymbol{C,g,e} \right)\boldsymbol{=C.c+\alpha g+\beta e+\gamma(g*e)+\varepsilon}$ (2)

where **g * e** refers to the element-wise product of two vectors. Let δ_e_ denote the concatenated effects for environmental factor **e**

$$\boldsymbol{\delta}_{\boldsymbol{e}}\boldsymbol{=}\binom{\boldsymbol{c}}{\begin{aligned} \boldsymbol{\alpha} \\ \boldsymbol{\beta} \\ \boldsymbol{\gamma} \end{aligned}}$$

One of the major problems with testing the γ parameter in this model is that if the environmental factor **e** is correlated with **y** and **g** the test may yield spurious interaction coefficients but only due to collider bias or biases well-established in secondary trait analysis.

In our simulation analysis rather than running the models above we perform the following:

$\left( \boldsymbol{y} | \boldsymbol{C,g,f} \right)\boldsymbol{=C.c+\alpha g+\beta f+\gamma(g*f)+\tau}$ (3)

where $\boldsymbol{f}$ relates to $\boldsymbol{y}$, $\boldsymbol{g}$ and ***C*** marginally as does $\boldsymbol{e}$ and has the same conditional distribution. In other words we create an artificial environmental variable that behaves marginally exactly as the real environmental variable $\boldsymbol{e}$.

In practice one can simulate ***f*** easily by regressing $\boldsymbol{e}$ on [$\boldsymbol{C,g,y}$] and add the fitted values to a random permutation of the residuals. This ensures that $\boldsymbol{f}$ and $\boldsymbol{e}$ have the same conditional expectations and same residual distributions.

**Testing for reverse causality**

To determine if the BMI genetic variants could influence BMI through primary effects on physical activity of diet related variables we investigated the association of each of the 69 BMI SNPs with physical activity, western diet and TV watching. We then plotted these against the published variant BMI-association to determine if any SNP was more associated with the obesogen than with BMI itself. To test the relationship between BMI and the obesogens we performed instrumental variable analysis (IVW) and a sensitivity analysis known to account for pleiotropy known as Egger Mendelian Randomisation (MR-Egger)^1^. The results of these analyses are plotted in supplementary figure 2.

**Additional sensitivity analyses**

There are (at least) three potential artefacts that can give rise to spurious GxE from stratified genetic analysis:

The first one arises when the ***variance of the outcome variable is dependent on the mean value of the environmental factor***. The simplest configuration that gives rise to this scenario is when the variance of the outcome variable depends on its mean (e.g. log normally, or Poisson distributed) and the environmental factor is correlated with the outcome. This is the case for BMI and most of obesogenic environmental factors. This not only results in heteroscedastic noise, but different outcome variability in the strata. Such artefact can be eliminated by robust linear regression and inverse normal quantile normalization of the outcome variable in each stratum.

***Splitting the sample into two groups with different environmental variability*** can also introduce spurious GxE association. This is typically the case when one stratifies the population using high threshold for the environmental variable. This artificially reduces the environmental variance in one of the groups and hence seemingly increases the observed genetic effect in that stratum. This problem can be reduced by splitting the sample such that the environmental variability is equal in the two groups. Alternatively, the environmental factor can be kept as a continuous variable and robust interaction model can be applied. In addition to using a continuous variable we also tested BMI genetic risk score – BMI associations when the high risk and low risk groups were defined as 75% people at the highest risk and 25% at low risk. These analyses demonstrated similar results with evidence for larger BMI GRS ~BMI effects in the high risk environment groups (Supplementary Table 10).

The third problem can arise due to index event bias. If the ***genetic variants are shared*** between the outcome and the environmental factor and the ***outcome is causally influencing the environmental factor***. In this case we observe classical index event bias for the estimation of the genetic effect in each stratum. This scenario can be excluded by testing the effect of GRS SNPs on the environmental factor to exclude pleiotropy. There was no correlation between the effect of individual BMI genetic variants on BMI and the effects of the individual BMI genetic variants on TV watching or other measures of the obesogenic environment (Pearson correlation coefficient comparing BMI and TV hour betas of 69 variants = -0.09).

In this study, risk factors in the interaction model - measures of the obesogenic environment – were associated with the outcome – BMI. In theory this problem could have created false positive evidence of interaction but the sensitivity analyses described above suggested this was not the case.

Supplementary figures


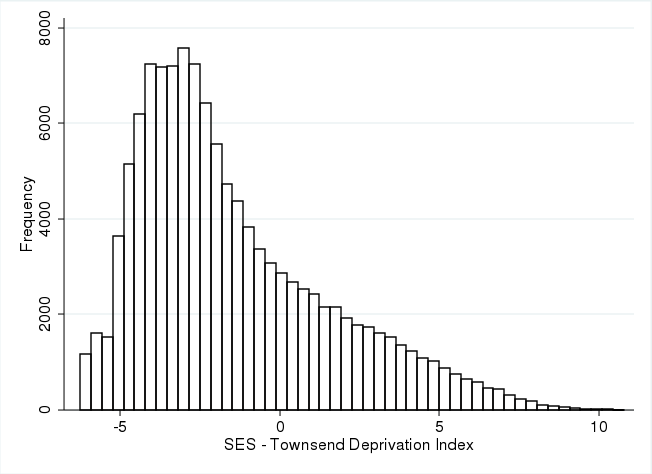
Supplementary Figure 1: Histogram showing the distribution of the Townsend Deprivation Index for the 119,733 individuals in the UK Biobank

Supplementary figure 2: The 69 BMI SNPs and their association with BMI in the primary GWAS^2^ plotted against their association with A) Physical activity determined by the International Physical Activity Questionnaire, B) TV watching and C) Western diet. The blue lines represent the standard instrumental variable association and the red lines represent a method of sensitivity analysis (Egger Mendelian Randomisation^1^)


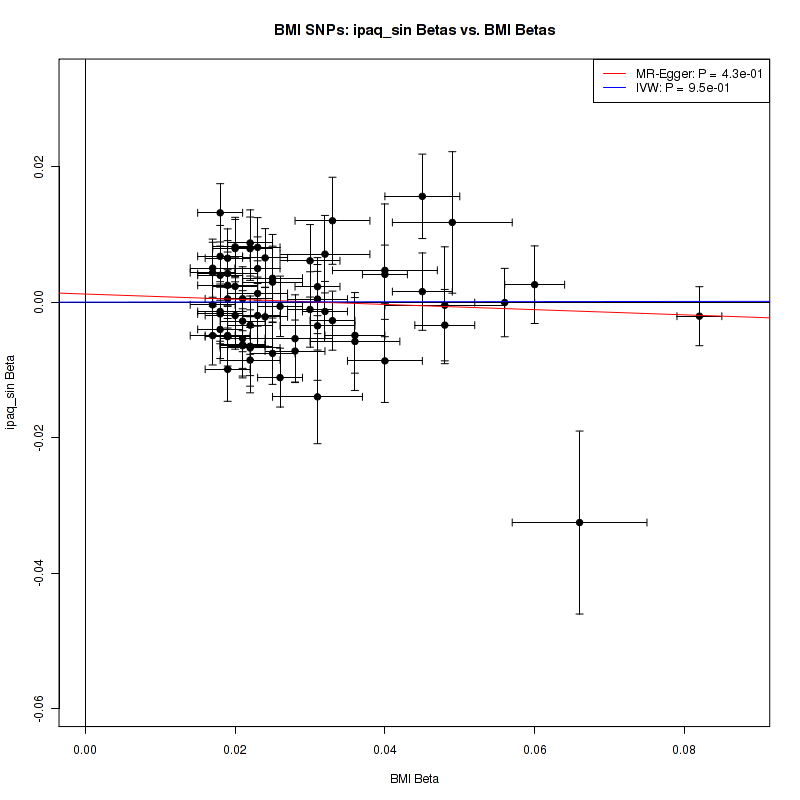


A


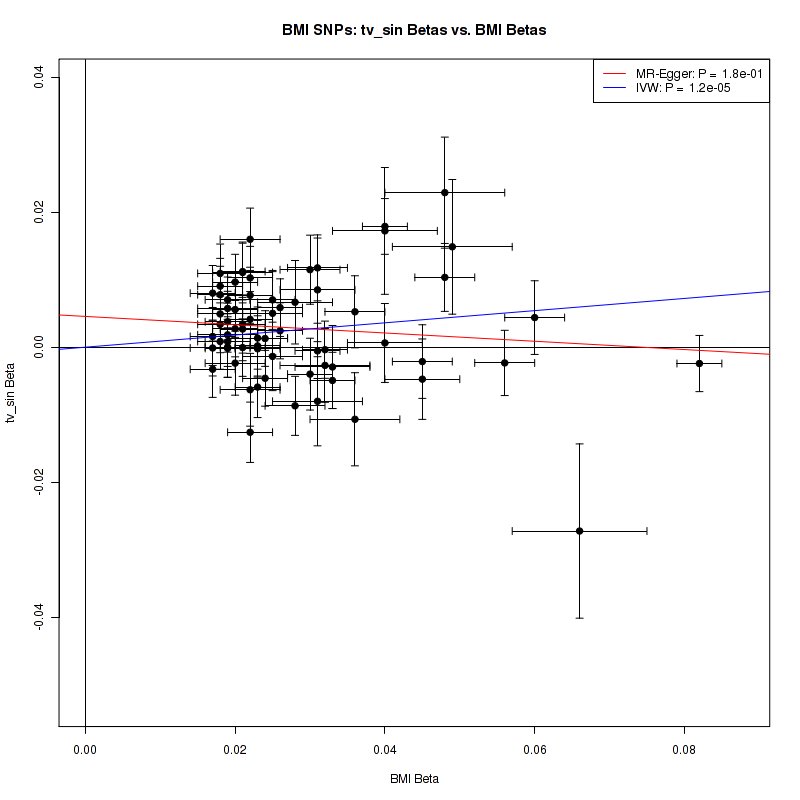


B


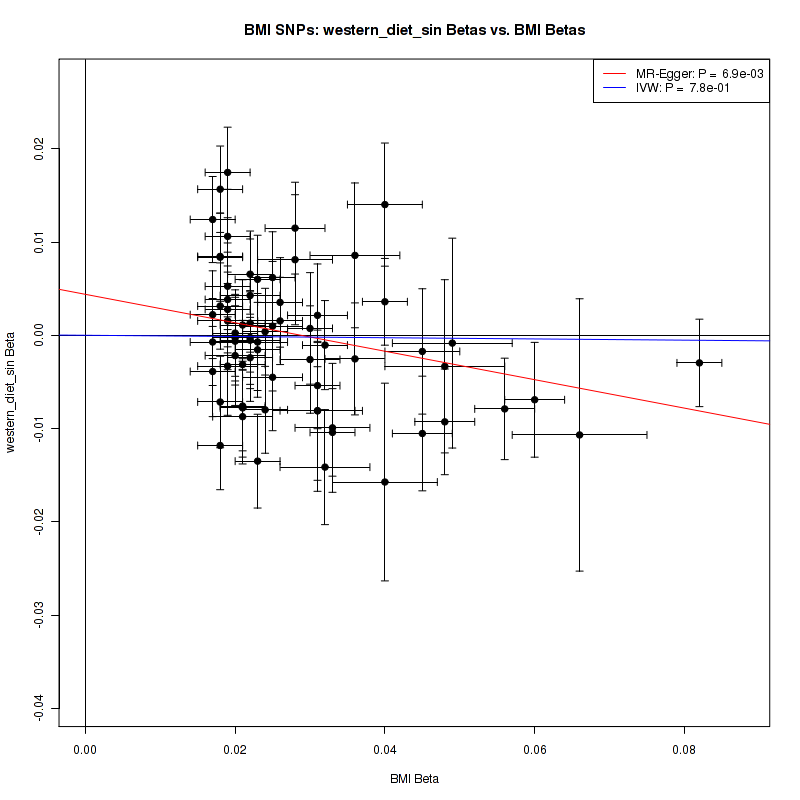


C

Supplementary figure 3: Scatter graph representing the relationship between mean BMI and the standard deviation of BMI for the 12 dichotomised obesogenic variables and the negative control sun protection use. Empty circles represent the low risk obesogenic variables and the solid circles represent the high risk obesogenic variables. The line of best fit indicates reasonable correlation (r^2^=0.78).

Supplementary Figure 4: Histograms representing the distribution of BMI in high TV watching and low TV watching groups. BMI represents raw BMI but effect sizes and p values are based on BMI adjusted for age, sex, ancestry principal components, assessment centre location and genotyping chip. BMI (INVERSE NORMAL) transforms the BMI residual variable to the inverse normal scale with a mean of zero and standard deviation of 1.
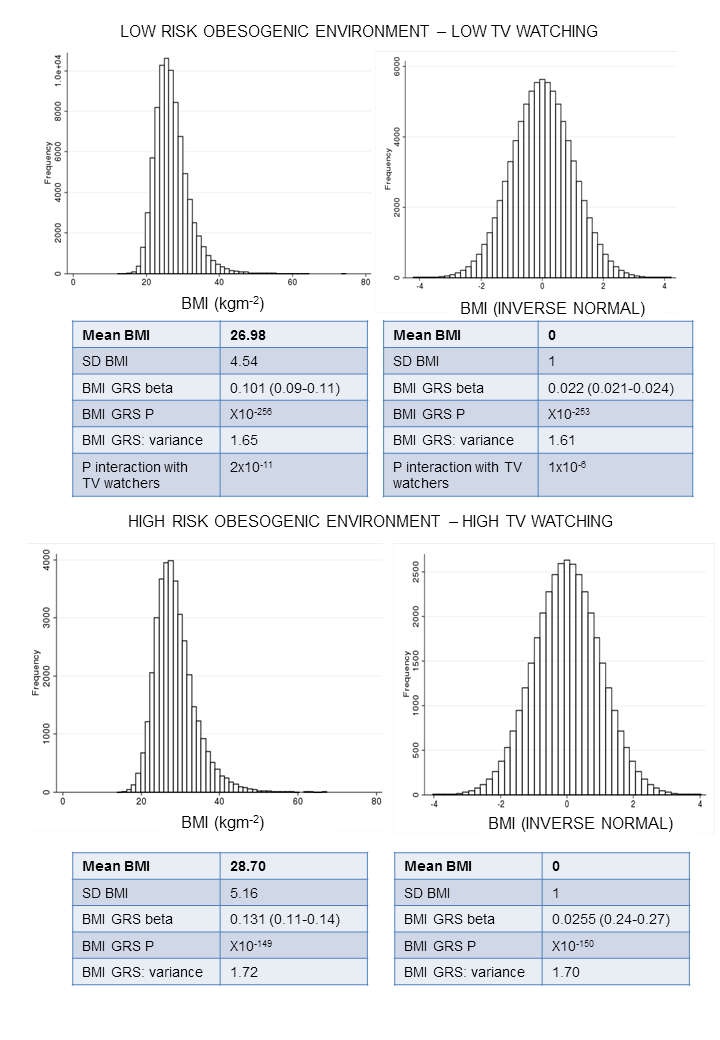


Supplementary Figure 5: QQ plot showing the observed TDI-BMI genetic interaction p-values from the 69 SNPs against the expected p-values

Supplementary Figure 6: Histograms of the –log_10_P values from the interaction analyses for the 100 groups generated by meta-heuristic sampling for A) Physical activity, B) TV watching, C) the composite score and D) fizzy drinks. The black dashed line represents the median p-value in the 100 random iterations and the red dashed line represents the interaction p-value obtained in the real data.


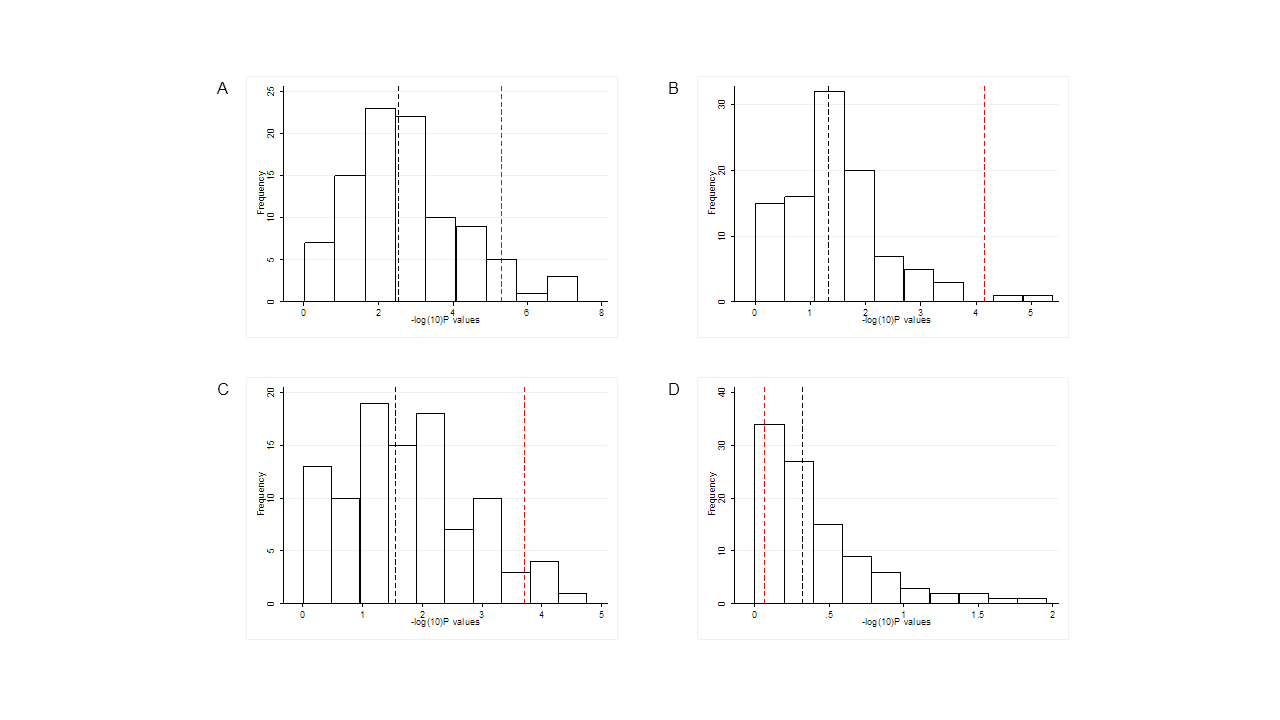


Supplementary figure 7: Histograms representing the interaction p-values for the 10,000 iterations of simulated dummy environments. The simulated environments were forced to have the same correlation with BMI as A) physical activity, B) TV watching, C) the composite score and D) fizzy drinks. The black line on the graphs represents the median interaction p-value in the simulations and the red line on graphs represents the real interaction p value.

A

B

b) ii

b) i

a) i

a) ii

C

D

Supplementary figure 8: Forest plot demonstrating the change in BMI on its natural scale (kgm^-2^), after correcting for age, sex and principle components per allele increase in BMI GRS for the different obesogenic environments.

Supplementary figure 9: Scatter graph representing the relationship between the beta values from the regression analyses for the BMI GRS and BMI and the standard deviation of BMI for the obesogenic variables. Empty circles represent the low risk obesogenic variables and the solid circles represent the high risk obesogenic variables. The line of best fit indicates reasonable correlation (r^2^=0.84).

**Supplementary Tables**

| Supplementary Table 1: Basic demographics of individuals included in the twelve obesogenic strata | | | | |
| --- | --- | --- | --- | --- |
| **Obesogenic category** | **Strata** | **N** | **Mean age at recruitment (SD)** | **Male N (%)** |
| Fizzy drink | All | 46,368 | 56.6 (7.8) | 21,927 (47.1) |
|  | None daily | 39,975 | 56.8 (7.7) | 18,327 (45.9) |
|  | >1 glass daily | 6,393 | 55.6 (8.0) | 3,537 (55.3) |
| Fried food intake | All | 46,368 | 56.6 (7.8) | 21,864 (47.2) |
|  | None daily | 31,821 | 56.6 (7.8) | 14,485 (45.5) |
|  | >1 meal daily | 14,547 | 56.7 (7.9) | 7,379 (50.7) |
| Percentage fat | All | 46,368 | 56.6 (7.8) | 21,864 (47.2) |
|  | Low risk | 23,194 | 56.9 (7.7) | 11,080 (47.8) |
|  | High risk | 23,174 | 56.4 (7.9) | 10,784 (46.5) |
| Percentage protein | All | 46,368 | 56.6 (7.8) | 21,864 (47.2) |
|  | Low risk | 23,188 | 56.5 (7.8) | 12,137 (52.3) |
|  | High risk | 23,180 | 56.7 (7.7) | 9,727 (42.0) |
| Western diet | All | 94,040 | 57.1 (7.9) | 44,636 (47.5) |
|  | Low risk | 47,027 | 56.8 (8.0) | 19,783 (42.1) |
|  | High risk | 47,013 | 57.3 (7.8) | 24,853 (52.9) |
| IPAQ | All | 109,142 | 56.9 (8.0) | 52,328 (47.9) |
|  | >1845 MET minutes per week | 54,573 | 56.9 (8.1) | 27,217 (49.9) |
|  | <1845 MET minutes per week | 54,569 | 56.9 (7.9) | 25,111 (46.0) |
| Sedentary time | All | 119,688 | 56.9 (7.9) | 56,668 (47.4) |
|  | <5 hours daily | 63,343 | 56.4 (8.0) | 25,281 (39.9) |
|  | >5 hours daily | 56,345 | 57.5 (7.8) | 31,387 (55.7) |
| TV watching | All | 118,836 | 56.9 (7.9) | 56,362 (47.4) |
|  | <4 hours daily | 82,022 | 56.0 (8.0) | 38,866 (47.4) |
|  | >4 hours daily | 36,814 | 59.0 (7.4) | 17,496 (47.5) |
| Minutes vigorous per week | All | 109,370 | 56.9 (8.0) | 52,432 (47.9) |
|  | >1 hour weekly | 35,242 | 55.8 (8.2) | 18,672 (53.0) |
|  | <1 hour weekly | 74,128 | 57.4 (7.8) | 33,760 (45.5) |
| Measured physical activity | All | 19,268 | 56.6 (7.7) | 8,815 (45.8) |
|  | Low risk | 9,632 | 55.0 (7.7) | 4,038 (41.9) |
|  | High risk | 9,636 | 58.2 (7.4) | 4,777 (49.6) |
| Socioeconomic position (TDI) | All | 119,733 | 56.9 (7.9) | 56,689 (47.4) |
|  | High SEP TDI<-2.294 | 59,872 | 57.4 (7.8) |  |
|  | Low SEP TDI>-2.294 | 59,861 | 56.5 (8.1) |  |
| Sun protection use | All | 119,068 | 56.9 (7.9) | 56,384 (47.4) |
|  | Usually or always use | 68,507 | 56.6 (7.9) | 25,641 (37.4) |
|  | Never or sometimes use | 50,561 | 57.4 (7.9) | 30,743 (60.8) |

All individuals are of white British descent with BMI and genetic data available (max n=119,688)

Supplementary table 2: Correlations between the ten obesogenic environment variables, Pearson correlation values are presented.

|  | TV watching | IPAQ | Minutes vigorous per week | Measured activity | Western diet | Percentage protein | Fizzy drink | Percentage fat | Fried food consumption | Socioeconomic position (TDI) | Sun protection |
| --- | --- | --- | --- | --- | --- | --- | --- | --- | --- | --- | --- |
|  |  |  |  |  |  |  |  |  |  |  |  |
| Sedentary time | 0.64 | -0.10 | 0.03 | -0.15 | 0.12 | 0.01 | 0.08 | 0.03 | 0.07 | 0.05 | -0.05 |
| TV watching | - | -0.09 | 0.03 | -0.15 | 0.09 | 0.04 | 0.05 | 0.03 | 0.06 | 0.12 | -0.02 |
| IPAQ | - | - | 0.46 | 0.17 | -0.01 | -0.02 | -0.01 | -0.05 | -0.02 | NS | 0.01 |
| Minutes vigorous per week | - | - | - | 0.07 | NS | NS | 0.01 | -0.01 | NS | 0.04 | -0.03 |
| Measured activity | - | - | - | - | -0.07 | -0.04 | NS | -0.02 | -0.02 | -0.03 | 0.03 |
| Western diet | - | - | - | - | - | 0.14 | 0.03 | 0.05 | 0.09 | -0.04 | -0.04 |
| Percentage protein | - | - | - | - | - | - | -0.10 | -0.08 | -0.12 | -0.04 | 0.08 |
| Fizzy drink | - | - | - | - | - | - | - | NS | 0.08 | 0.03 | -0.04 |
| Percentage fat | - | - | - | - | - | - | - | - | 0.14 | 0.03 | -0.06 |
| Fried food consumption | - | - | - | - | - | - | - | - | - | 0.01 | -0.04 |
| Socioeconomic position (TDI) | - | - | - | - | - | - | - | - | - | - | -0.07 |

NS – association between the two obesogenic measures p>0.05.

Supplementary table 3: Associations between twelve continuous or ordinal obesogenic factors and a range of demographic factors.

| Demographic | Regression coefficient (95% CI) representing change in obesogenic factor per unit change in demographic factor | P |
| --- | --- | --- |
| Physical Activity Based on IPAQ | | |
| Age | 0.00 (-0.00, 0.00) | 0.74 |
| Male sex | 0.10 (0.08, 0.11) | <1E-15 |
| BMI | -0.04 (-0.04, -0.04) | <1E-15 |
| SEP* | -0.00 (-0.01, 0.00) | 0.28 |
| Never smoking | 0.00 (-0.01, 0.02) | 0.7 |
| Type 2 diabetes (BMI adjusted) | -0.17 (-0.21, -0.13) | 4E-17 |
| Sedentary time | | |
| Age | 0.005 (0.003, 0.006) | 7E-08 |
| Male sex | 0.92 (0.90, 0.95) | <1E-15 |
| Body mass index | 0.11 (0.10, 0.11) | <1E-15 |
| SEP* | 0.04 (0.03, 0.04) | 4E-63 |
| Never smoking | -0.67 (-0.71, -0.62) | 2E-197 |
| Type 2 diabetes | 0.86 (0.79, 0.93) | 3E-114 |
| Physical activity | -0.23 (-0.24, -0.22) | <1E-15 |
| TV watching | | |
| Age | 0.043 (0.042, 0.045) | <1E-15 |
| Male sex | 0.017 (-0.004, 0.037) | 0.11 |
| Body mass index | 0.076 (0.074, 0.078) | <1E-15 |
| SEP* | 0.082 (0.079, 0.086) | <1E-15 |
| Never smoking | -0.77 (-0.81, -0.74) | <1E-15 |
| Type 2 diabetes | 0.73 (0.67, 0.78) | <1E-15 |
| Physical activity | -0.13 (-0.14, -0.13) | <1E-15 |
| Vigorous minutes per week | | |
| Age | -1.2 (-1.3, -1.0) | <1E-15 |
| Male sex | 33.3 (31.2, 35.3) | <1E-15 |
| Body mass index | -2.3 (-2.5, -2.1) | <1E-15 |
| SEP* | 0.29 (-0.06, 0.63) | 0.11 |
| Never smoking | 6.2 (2.8, 9.6) | 0.0004 |
| Type 2 diabetes | -20.8 (-15.0, -26.7) | 3E-12 |
| Physical activity | 70.7 (69.9, 71.5) | <1E-15 |
| Measured physical activity | | |
| Age | -137001 (-146819, -127183) | <1E-15 |
| Male sex | -454455 (-606000, -302910) | 4E-9 |
| Body mass index | -248909 (-265111, -232707) | <1E-15 |
| SEP* | -96115 (-123780, -68450) | 1E-11 |
| Never smoking | 1590365 (1252624, 1928107) | <1E-15 |
| Type 2 diabetes | -2579057 (-3071767, -2086348) | <1E-15 |
| Physical activity | 889837 (819458, 960217) | <1E-15 |
| Western diet | | |
| Age | 0.01 (0.01, 0.01) | 7E-71 |
| Male sex | 0.28 (0.27, 0.29) | <1E-15 |
| Body mass index | 0.02 (0.02, 0.03) | 1E-301 |
| SEP* | -0.01 (-0.02, -0.01) | 5E-33 |
| Never smoking | -0.10 (0.08, 0.12) | 3E-19 |
| Type 2 diabetes | 0.15 (0.12, 0.19) | 5E-18 |
| Physical activity | -0.02 (-0.02, -0.01) | 1E-07 |
| Fizzy drink** | | |
| Age | -0.02 (-0.03, -0.02) | 3E-37 |
| Male sex | 0.41 (0.36, 0.46) | 5E-52 |
| Body mass index | 0.03 (0.03, 0.04) | 2E-34 |
| SEP* | 0.02 (0.01, 0.03) | 5E-04 |
| Never smoking | -0.21 (-0.31, -0.12) | 9E-06 |
| Type 2 diabetes | 0.05 (-0.11, 0.21) | 0.55 |
| Physical activity | -0.04 (-0.06, -0.01) | 0.003 |
| Fried food** | | |
| Age | 0.00 (-0.00, 0.00) | 0.28 |
| Male sex | 0.25 (0.21, 0.29) | 2E-37 |
| Body mass index | 0.01 (0.01, 0.01) | 4E-07 |
| SEP* | 0.00 (-0.01, 0.00) | 0.29 |
| Never smoking | 0.11 (0.04, 0.18) | 2E-03 |
| Type 2 diabetes | 0.05 (-0.07, 0.17) | 0.41 |
| Physical activity | -0.03 (-0.05, -0.01) | 9E-04 |
| Percentage fat | | |
| Age | -0.001 (-0.001, -0.001) | 1E-15 |
| Male sex | -0.01 (-0.01, -0.00) | 1E-04 |
| Body mass index | 0.002 (0.001, 0.002) | 4E-17 |
| SEP* | 0.002 (0.001, 0.002) | 8E-08 |
| Never smoking | -0.03 (-0.04, -0.03) | 2E-26 |
| Type 2 diabetes | 0.04 (0.03, 0.05) | 1E-11 |
| Physical activity | -0.01 (-0.01, -0.01) | 5E-22 |
| Percentage protein | | |
| Age | 0.0003 (0.0001, 0.0005) | 0.01 |
| Male sex | -0.05 (-0.05, -0.05) | 2E-133 |
| Body mass index | 0.01 (0.01, 0.01) | 5E-118 |
| SEP* | -0.003 (-0.004, -0.002) | 7E-14 |
| Never smoking | 0.03 (0.03, 0.04) | 4E-18 |
| Type 2 diabetes | 0.05 (0.04, 0.06) | 1E-15 |
| Physical activity | -0.003 (-0.005, -0.001) | 0.003 |
| Composite Score | | |
| Age | 0.018 (0.017, 0.019) | <1E-15 |
| Male sex | 0.130 (0.117, 0.143) | <1E-15 |
| Body mass index | 0.060 (0.059, 0.061) | <1E-15 |
| SEP* | 0.024 (0.022, 0.027) | <1E-15 |
| Never smoking | -0.192 (-0.205, -0.178) | 6E-173 |
| Type 2 diabetes | 0.506 (0.469, 0.542) | 2E-158 |
| Physical activity | -0.448 (-0.453, -0.443) | <1E-15 |
| Townsend deprivation index | | |
| Age | -0.024 (-0.026, -0.022) | <1E-15 |
| Male sex | 0.071 (0.037, 0.105) | 4E-5 |
| Body mass index | 0.057 (0.053, 0.060) | <1E-15 |
| Never smoking | -2.00 (-2.05, -1.95) | <1E-15 |
| Type 2 diabetes | 0.79 (0.69, 0.88) | <1E-15 |
| Physical activity | -0.008 (-0.024, 0.007) | 0.28 |
|  | Sun protection use** |  |
| Age | -0.008 (-0.009, -0.006) | <1E-15 |
| Male sex | -0.96 (-0.99, -0.94) | <1E-15 |
| Body mass index | -0.009 (-0.011, -0.007) | 8E-15 |
| SEP* | -0.044 (-0.047, -0.040) | <1E-15 |
| Never smoking | 0.50 (0.47, 0.54) | <1E-15 |
| Type 2 diabetes | -0.21 (-0.27, -0.15) | 5E-12 |
| Physical activity | 0.038 (0.029, 0.048) | 8E-15 |

All models adjusted for age and sex

***** Socioeconomic position (SEP) is defined by the Townsend deprivation index

** Associations were investigated using ordinal regression due to the distribution of the dependent variable.

| Supplementary table 4: Summary of genetic variants previously identified as associated with BMI at genome wide significance | | | | | | | | | | |
| --- | --- | --- | --- | --- | --- | --- | --- | --- | --- | --- |
| **Trait** | **SNP** | **Locus** | **Exclude from score** | **Reason for exclusion** | **Trait raising allele** | **Trait lowering allele** | **Directly genotyped or Imputed** | **Imputation quality** | **Beta representing SD change in BMI for each SNP in UK Biobank data** | **P value** |
| BMI | rs1000940 | *RABEP1* | No | NA | G | A | Imputed | 0.99624 | 0.011 (0.004) | 0.016 |
| BMI | rs10132280 | *STXBP6* | No | NA | C | A | Imputed | 0.97496 | 0.020 (0.005) | 1x10^-5^ |
| BMI | rs1016287 | *FLJ30838* | No | NA | T | C | Imputed | 0.99411 | 0.019 (0.004) | 2x10^-5^ |
| BMI | rs10182181 | *ADCY3* | No | NA | G | A | Imputed | 0.99521 | 0.033 (0.004) | 1x10^-15^ |
| BMI | rs10733682 | *LMX1B* | No | NA | A | G | Imputed | 0.9576 | 0.019 (0.004) | 6x10^-6^ |
| BMI | rs10938397 | *GNPDA2* | No | NA | G | A | Imputed | 1 | 0.030 (0.004) | 6x10^-13^ |
| BMI | rs10968576 | *LINGO2* | No | NA | G | A | Imputed | 1 | 0.024 (0.004) | 7x10^-8^ |
| BMI | rs11030104 | *BDNF* | Yes | BMI-raising allele also associated with regular smoking (which itself has a causal effect on BMI in opposite direction) | A | G | Imputed | 0.99931 | NA | NA |
| BMI | rs11057405 | *CLIP1* | No | NA | G | A | Imputed | 1 | 0.030 (0.007) | 5x10^-6^ |
| BMI | rs11126666 | *KCNK3* | No | NA | A | G | Imputed | 0.99485 | 0.002 (0.005) | 0.71 |
| BMI | rs11165643 | *PTBP2* | No | NA | T | C | Imputed | 0.99575 | 0.016 (0.004) | 1x10^-4^ |
| BMI | rs11191560 | *NT5C2* | No | NA | C | T | Imputed | 0.99989 | 0.026 (0.008) | 7x10^-4^ |
| BMI | rs11583200 | *ELAVL4* | No | NA | C | T | Imputed | 0.98728 | 0.019 (0.004) | 8x10^-6^ |
| BMI | rs1167827 | *HIP1* | No | NA | G | A | Imputed | 1 | 0.020 (0.004) | 2x10^-6^ |
| BMI | rs11688816 | *EHBP1* | No | NA | G | A | Imputed | 0.98096 | 0.014 (0.004) | 9x10^-4^ |
| BMI | rs11727676 | *HHIP* | No | NA | T | C | Imputed | 1 | -0.003 (0.007) | 0.66 |
| BMI | rs11847697 | *PRKD1* | No | NA | T | C | Imputed | 1 | 0.014 (0.010) | 0.17 |
| BMI | rs12286929 | *CADM1* | No | NA | G | A | Imputed | 0.99124 | 0.010 (0.004) | 0.012 |
| BMI | rs12401738 | *FUBP1* | No | NA | A | G | Imputed | 0.99528 | 0.012 (0.004) | 0.003 |
| BMI | rs12429545 | *OLFM4* | No | NA | A | G | Imputed | 0.97759 | 0.027 (0.006) | 8x10^-6^ |
| BMI | rs12446632 | *GPRC5B* | No | NA | G | A | Imputed | 0.99978 | 0.028 (0.006) | 2x10^-6^ |
| BMI | rs12566985 | *FPGT-TNNI3K* | No | NA | G | A | Imputed | 0.9947 | 0.011 (0.004) | 0.006 |
| BMI | rs12885454 | *PRKD1* | No | NA | C | A | Imputed | 0.99569 | 0.015 (0.004) | 5x10^-4^ |
| BMI | rs12940622 | *RPTOR* | No | NA | G | A | Imputed | 0.99796 | 0.017 (0.004) | 6x10^-5^ |
| BMI | rs13021737 | *TMEM18* | No | NA | G | A | Imputed | 0.99072 | 0.059 (0.005) | 9x10^-27^ |
| BMI | rs13078960 | *CADM2* | No | NA | G | T | Imputed | 0.9915 | 0.024 (0.005) | 3x10^-6^ |
| BMI | rs13107325 | *SLC39A8* | Yes | Missense Ala/Thr polymorphism located in exon 7 of SLC39A8, which encodes a zinc transporter that also transports cadmium and manganese. It is also associated with BP and HDL levels, and presumably these and the BMI effect are secondary to the metal ion transport variation. | T | C | Imputed | 1 | NA | NA |
| BMI | rs13191362 | *PARK2* | No | NA | A | G | Imputed | 0.98973 | 0.026 (0.006) | 3x10^-5^ |
| BMI | rs1516725 | *ETV5* | No | NA | C | T | Imputed | 0.99495 | 0.032 (0.006) | 1x10^-7^ |
| BMI | rs1528435 | *UBE2E3* | No | NA | T | C | Imputed | 0.99738 | 0.014 (0.004) | 7x10^-4^ |
| BMI | rs1558902 | *FTO* | No | NA | A | T | Imputed | 0.99914 | 0.077 (0.004) | 2x10^-75^ |
| BMI | rs16851483 | *RASA2* | No | NA | T | G | Imputed | 0.99906 | 0.028 (0.008) | 7x10^-4^ |
| BMI | rs16951275 | *MAP2K5* | No | NA | T | C | Imputed | 0.99819 | 0.032 (0.005) | 4x10^-11^ |
| BMI | rs17001654 | *SCARB2* | Yes | SNP not in HWE | G | C | Imputed | 0.9483 | NA | NA |
| BMI | rs17024393 | *GNAT2* | No | NA | C | T | Imputed | 0.98934 | 0.074 (0.013) | 1x10^-8^ |
| BMI | rs17094222 | *HIF1AN* | No | NA | C | T | Imputed | 0.96874 | 0.013 (0.005) | 0.009 |
| BMI | rs17405819 | *HNF4G* | No | NA | T | C | Imputed | 0.99793 | 0.014 (0.004) | 0.001 |
| BMI | rs17724992 | *PGPEP1* | No | NA | A | G | Imputed | 0.98342 | 0.023 (0.005) | 1x10^-6^ |
| BMI | rs1808579 | *C18orf8* | No | NA | C | T | Imputed | 0.99797 | 0.022 (0.004) | 2x10^-7^ |
| BMI | rs1928295 | *TLR4* | No | NA | T | C | Imputed | 0.99998 | 0.010 (0.004) | 0.016 |
| BMI | rs2033529 | *TDRG1* | Yes | SNP not available | G | A | NA | NA | NA | NA |
| BMI | rs2033732 | *RALYL* | No | NA | C | T | Imputed | 1 | 0.002 (0.005) | 0.67 |
| BMI | rs205262 | *C6orf106* | No | NA | G | A | Imputed | 0.9968 | 0.028 (0.005) | 1x10^-9^ |
| BMI | rs2075650 | *TOMM40* | Yes | SNP not in HWE | A | G | Imputed | 0.9865 | NA | NA |
| BMI | rs2112347 | *POC5* | No | NA | T | G | Imputed | 1 | 0.026 (0.004) | 6x10^-10^ |
| BMI | rs2121279 | *LRP1B* | No | NA | T | C | Imputed | 0.98723 | 0.006 (0.006) | 0.37 |
| BMI | rs2176598 | *HSD17B12* | No | NA | T | C | Imputed | 1 | 0.023 (0.005) | 1x10^-6^ |
| BMI | rs2207139 | *TFAP2B* | No | NA | G | A | Imputed | 0.9989 | 0.038 (0.005) | 2x10^-12^ |
| BMI | rs2245368 | *PMS2L11* | No | NA | C | T | Imputed | 1 | 0.022 (0.005) | 8x10^-5^ |
| BMI | rs2287019 | *QPCTL* | No | NA | C | T | Imputed | 0.97852 | 0.035 (0.005) | 1x10^-10^ |
| BMI | rs2365389 | *FHIT* | No | NA | C | T | Imputed | 0.99305 | 0.029 (0.004) | 3x10^-12^ |
| BMI | rs2650492 | *SBK1* | No | NA | A | G | Imputed | 0.98144 | 0.019 (0.005) | 4x10^-5^ |
| BMI | rs2820292 | *NAV1* | No | NA | C | A | Imputed | 1 | 0.019 (0.004) | 4x10^-6^ |
| BMI | rs29941 | *KCTD15* | No | NA | G | A | Imputed | 1 | 0.018 (0.004) | 5x10^-5^ |
| BMI | rs3101336 | *NEGR1* | No | NA | C | T | Imputed | 1 | 0.027 (0.004) | 1x10^-10^ |
| BMI | rs3736485 | *DMXL2* | No | NA | A | G | Imputed | 0.98728 | 0.011 (0.004) | 0.006 |
| BMI | rs3810291 | *ZC3H4* | No | NA | A | G | Imputed | 1 | 0.028 (0.004) | 2x10^-10^ |
| BMI | rs3817334 | *MTCH2* | No | NA | T | C | Imputed | 1 | 0.031 (0.004) | 1x10^-13^ |
| BMI | rs3849570 | *GBE1* | No | NA | A | C | Imputed | 0.99509 | 0.011 (0.004) | 0.008 |
| BMI | rs3888190 | *ATP2A1* | Yes | Associated with lots of other traits and is a big haplotype | A | C | Imputed | 0.99808 | NA | NA |
| BMI | rs4256980 | *TRIM66* | No | NA | G | C | Imputed | 0.99283 | 0.021 (0.004) | 2x10^-6^ |
| BMI | rs4740619 | *C9orf93* | No | NA | T | C | Imputed | 0.99762 | 0.017 (0.004) | 6x10^-5^ |
| BMI | rs543874 | *SEC16B* | No | NA | G | A | Imputed | 1 | 0.049 (0.005) | 3x10^-22^ |
| BMI | rs6477694 | *EPB41L4B* | No | NA | C | T | Imputed | 0.99022 | 0.008 (0.004) | 0.07 |
| BMI | rs6567160 | *MC4R* | No | NA | C | T | Imputed | 0.99663 | 0.054 (0.005) | 1x10^-28^ |
| BMI | rs657452 | *AGBL4* | No | NA | A | G | Imputed | 0.98709 | 0.014 (0.004) | 8x10^-4^ |
| BMI | rs6804842 | *RARB* | No | NA | G | A | Imputed | 0.98778 | 0.009 (0.004) | 0.032 |
| BMI | rs7138803 | *BCDIN3D* | No | NA | A | G | Imputed | 1 | 0.034 (0.004) | 1x10^-15^ |
| BMI | rs7141420 | *NRXN3* | No | NA | T | C | Imputed | 0.98379 | 0.019 (0.004) | 7x10^-6^ |
| BMI | rs7243357 | *GRP* | No | NA | T | G | Imputed | 0.98998 | 0.012 (0.005) | 0.021 |
| BMI | rs758747 | *NLRC3* | No | NA | T | C | Imputed | 0.97187 | 0.014 (0.005) | 0.002 |
| BMI | rs7599312 | *ERBB4* | No | NA | G | A | Imputed | 0.97294 | 0.019 (0.005) | 4x10^-5^ |
| BMI | rs7899106 | *GRID1* | No | NA | G | A | Imputed | 0.98612 | 0.023 (0.009) | 0.014 |
| BMI | rs9400239 | *FOXO3* | No | NA | C | T | Imputed | 0.99206 | 0.017 (0.005) | 2x10^-4^ |
| BMI | rs9581854 | *MTIF3* | No | NA | T | C | Imputed | 0.98643 | 0.015 (0.005) | 0.006 |
| BMI | rs9925964 | *KAT8* | Yes | SNP not in HWE | A | G | Imputed | 1 | NA | NA |

**Supplementary table 5**: Association between the various obesogenic measures and the BMI genetic risk score

| **Obesogenic factor** | **N** | **Beta (95%) representing an SD change in obesogenic factor per allele increase in the BMI weighted allele score** | **P** |
| --- | --- | --- | --- |
| Sedentary time | 119688 | 0.002 (0.001, 0.003) | 0.004 |
| TV watching | 118836 | 0.002 (0.001, 0.003) | 1x10^-5^ |
| IPAQ | 109142 | -0.0001 (-0.001, 0.001) | 0.85 |
| Minutes of vigorous activity | 109370 | 0.001 (0.000, 0.002) | 0.044 |
| Total activity | 19268 | -0.004 (-0.007, -0.001) | 0.005 |
| Western diet | 94040 | -0.001 (-0.002, 0.000) | 0.21 |
| Percentage protein | 46368 | 0.005 (0.004, 0.007) | 5x10^-10^ |
| Fizzy drink | 46368 | -0.005 (-0.010, -0.000) | 0.04 |
| Percentage fat | 46368 | 0.000 (-0.000, 0.001) | 0.16 |
| Fried food consumption | 46368 | -0.002 (-0.003, -0.001) | 0.002 |
| Composite score | 86549 | 0.002 (0.001, 0.003) | 0.006 |
| SEP (TDI) | 119,733 | 0.001 (0.000, 0.002) | 0.022 |
| Sun protection | 119,068 | -0.0006 (-0.0016, 0.0004) | 0.23 |

| **Supplementary Table 6:** Differences in BMI by BMI genetic risk score decile (kgm^2^) and by allele (inverse normalised scale) for a) Townsend deprivation index split at the median and b) Townsend deprivation index split at the approximate UK average deprivation value. Interaction p-values are calculated using the binary TDI variable for both to enable comparison. | | | | | | | | | | | |
| --- | --- | --- | --- | --- | --- | --- | --- | --- | --- | --- | --- |
| **Trait** | **Obesogenic category** | **N** | **BMI (SD)** | **BMI difference in 10% lowest genetic risk** | **BMI difference in 10% highest genetic risk** | **Per-allele Beta** | **SE** | **P association** | **P interaction*** | **P Interaction Robust**** |  |
| Townsend Deprivation Index (natural scale) | High SES TDI<-2.294 | 59,872 | 27.20  (4.47) |  |  | 0.022 | 0.001 | <1x10^-15^ | **4x10^-6^** | **5x10^-6^** |  |
|  | Low SES TDI>-2.294 | 59,861 | 27.87  (5.13) | +0.35 kgm^-2^ | +0.92 kgm^-2^ | 0.025 | 0.001 | <1x10^-15^ |  |  |  |
| Townsend Deprivation Index (natural scale) | High SES | 84,526 | 27.30  (4.56) |  |  | 0.022 | 0.001 | <1x10^-15^ | **9x10^-9^** | **6x10^-8^** |  |
|  | Low SES | 35,357 | 28.11  (5.37) | +0.42 kgm^-2^ | +1.06 kgm^-2^ | 0.027 | 0.001 | <1x10^-15^ |  |  |  |

**Supplementary Table 7:** BMI genetic risk score association with BMI for different age groups in the UK Biobank. The interaction effect was then investigated for TDI in the three age groups and the P values for normal and robust models are presented.

| Age group | N | Beta for BMI GRS against BMI | SE | P | Variance explained (%) | TDI Pinteraction | TDI Pinteraction robust |
| --- | --- | --- | --- | --- | --- | --- | --- |
| 40-49 | 25658 | 0.028 | 0.001 | <1x10^-15^ | 2 | 9x10^-5^ | 3x10^-4^ |
| 50-59 | 40131 | 0.025 | 0.001 | <1x10^-15^ | 1.7 | 3x10^-5^ | 1x10^-4^ |
| 60-73 | 53944 | 0.020 | 0.0008 | <1x10^-15^ | 1.2 | 6x10^-4^ | 0.001 |

| **Supplementary table 8:** Individual SNP associations with BMI in high and low Townsend deprivation index groups | | | | | | |
| --- | --- | --- | --- | --- | --- | --- |
| **SNP** | **Obesogenic category** | **Beta** | **SE** | **P association** | **P interaction** | **P interaction robust** |
| rs1000940 | Low SES | 0.001 | 0.006 | 0.90 | 0.10 | 0.13 |
|  | High SES | 0.020 | 0.006 | 0.002 |  |  |
| rs10132280 | Low SES | 0.020 | 0.006 | 0.002 | 0.30 | 0.33 |
|  | High SES | 0.021 | 0.006 | 8x10^-4^ |  |  |
| rs1016287 | Low SES | 0.007 | 0.006 | 0.30 | 9x10^-4^ | 0.002 |
|  | High SES | 0.031 | 0.006 | 1x10^-6^ |  |  |
| rs10182181 | Low SES | 0.035 | 0.006 | 2x10^-9^ | 0.50 | 0.53 |
|  | High SES | 0.032 | 0.006 | 3x10^-8^ |  |  |
| rs10733682 | Low SES | 0.021 | 0.006 | 4x10^-4^ | 0.99 | 0.99 |
|  | High SES | 0.017 | 0.006 | 0.004 |  |  |
| rs10938397 | Low SES | 0.024 | 0.006 | 4x10^-5^ | 0.013 | 0.02 |
|  | High SES | 0.037 | 0.006 | 4x10^-10^ |  |  |
| rs10968576 | Low SES | 0.024 | 0.006 | 1x10^-4^ | 0.49 | 0.51 |
|  | High SES | 0.024 | 0.006 | 1x10^-4^ |  |  |
| rs11057405 | Low SES | 0.031 | 0.009 | 8x10^-4^ | 0.61 | 0.63 |
|  | High SES | 0.030 | 0.009 | 0.001 |  |  |
| rs11126666 | Low SES | 0.001 | 0.007 | 0.93 | 0.89 | 0.90 |
|  | High SES | 0.004 | 0.007 | 0.53 |  |  |
| rs11165643 | Low SES | 0.017 | 0.006 | 0.003 | 0.47 | 0.49 |
|  | High SES | 0.014 | 0.006 | 0.014 |  |  |
| rs11191560 | Low SES | 0.037 | 0.011 | 6x10^-4^ | 0.49 | 0.71 |
|  | High SES | 0.019 | 0.011 | 0.08 |  |  |
| rs11583200 | Low SES | 0.020 | 0.006 | 0.001 | 0.54 | 0.56 |
|  | High SES | 0.017 | 0.006 | 0.005 |  |  |
| rs1167827 | Low SES | 0.019 | 0.006 | 0.001 | 0.93 | 0.94 |
|  | High SES | 0.019 | 0.006 | 9x10^-4^ |  |  |
| rs11688816 | Low SES | 0.019 | 0.006 | 0.001 | 0.59 | 0.61 |
|  | High SES | 0.009 | 0.006 | 0.13 |  |  |
| rs11727676 | Low SES | 0.001 | 0.010 | 0.95 | 0.50 | 0.52 |
|  | High SES | -0.008 | 0.010 | 0.41 |  |  |
| rs11847697 | Low SES | 0.007 | 0.014 | 0.61 | 0.25 | 0.28 |
|  | High SES | 0.019 | 0.014 | 0.17 |  |  |
| rs12286929 | Low SES | 0.011 | 0.006 | 0.07 | 0.94 | 0.95 |
|  | High SES | 0.010 | 0.006 | 0.10 |  |  |
| rs12401738 | Low SES | 0.008 | 0.006 | 0.15 | 0.46 | 0.49 |
|  | High SES | 0.015 | 0.006 | 0.011 |  |  |
| rs12429545 | Low SES | 0.029 | 0.009 | 9x10^-4^ | 0.27 | 0.30 |
|  | High SES | 0.028 | 0.009 | 0.001 |  |  |
| rs12446632 | Low SES | 0.029 | 0.008 | 5x10^-4^ | 0.45 | 0.48 |
|  | High SES | 0.027 | 0.008 | 0.001 |  |  |
| rs12566985 | Low SES | 0.012 | 0.006 | 0.044 | 0.21 | 0.24 |
|  | High SES | 0.010 | 0.006 | 0.08 |  |  |
| rs12885454 | Low SES | 0.017 | 0.006 | 0.004 | 0.33 | 0.35 |
|  | High SES | 0.014 | 0.006 | 0.017 |  |  |
| rs12940622 | Low SES | 0.021 | 0.006 | 3x10^-4^ | 0.72 | 0.74 |
|  | High SES | 0.013 | 0.006 | 0.024 |  |  |
| rs13021737 | Low SES | 0.049 | 0.008 | 1x10^-10^ | 0.20 | 0.23 |
|  | High SES | 0.067 | 0.008 | 6x10^-18^ |  |  |
| rs13078960 | Low SES | 0.025 | 0.007 | 4x10^-4^ | 0.99 | 0.99 |
|  | High SES | 0.024 | 0.007 | 0.001 |  |  |
| rs13191362 | Low SES | 0.026 | 0.009 | 0.003 | 0.39 | 0.43 |
|  | High SES | 0.023 | 0.009 | 0.008 |  |  |
| rs1516725 | Low SES | 0.034 | 0.008 | 5x10^-5^ | 0.67 | 0.69 |
|  | High SES | 0.029 | 0.008 | 7x10^-4^ |  |  |
| rs1528435 | Low SES | 0.014 | 0.006 | 0.023 | 0.29 | 0.32 |
|  | High SES | 0.014 | 0.006 | 0.015 |  |  |
| rs1558902 | Low SES | 0.072 | 0.006 | 5x10^-34^ | 0.006 | 0.010 |
|  | High SES | 0.081 | 0.006 | 3x10^-43^ |  |  |
| rs16851483 | Low SES | 0.010 | 0.012 | 0.38 | 0.22 | 0.26 |
|  | High SES | 0.045 | 0.012 | 1x10^-4^ |  |  |
| rs16951275 | Low SES | 0.036 | 0.007 | 1x10^-7^ | 0.23 | 0.26 |
|  | High SES | 0.028 | 0.007 | 6x10^-5^ |  |  |
| rs17024393 | Low SES | 0.055 | 0.018 | 0.003 | 0.091 | 0.11 |
|  | High SES | 0.091 | 0.018 | 6x10^-7^ |  |  |
| rs17094222 | Low SES | 0.012 | 0.007 | 0.09 | 0.25 | 0.29 |
|  | High SES | 0.014 | 0.007 | 0.05 |  |  |
| rs17405819 | Low SES | 0.013 | 0.006 | 0.046 | 0.47 | 0.50 |
|  | High SES | 0.018 | 0.006 | 0.05 |  |  |
| rs17724992 | Low SES | 0.017 | 0.007 | 0.011 | 0.68 | 0.70 |
|  | High SES | 0.027 | 0.007 | 5x10^-5^ |  |  |
| rs1808579 | Low SES | 0.019 | 0.006 | 9x10^-4^ | 0.21 | 0.24 |
|  | High SES | 0.023 | 0.006 | 9x10^-5^ |  |  |
| rs1928295 | Low SES | 0.001 | 0.006 | 0.83 | 0.24 | 0.27 |
|  | High SES | 0.018 | 0.006 | 0.002 |  |  |
| rs2033732 | Low SES | 0.011 | 0.007 | 0.11 | 0.07 | 0.09 |
|  | High SES | -0.006 | 0.007 | 0.33 |  |  |
| rs205262 | Low SES | 0.031 | 0.007 | 3x10^-6^ | 0.76 | 0.77 |
|  | High SES | 0.025 | 0.007 | 1x10^-4^ |  |  |
| rs2112347 | Low SES | 0.029 | 0.006 | 2x10^-6^ | 0.54 | 0.57 |
|  | High SES | 0.024 | 0.006 | 7x10^-5^ |  |  |
| rs2121279 | Low SES | 0.009 | 0.009 | 0.33 | 0.50 | 0.53 |
|  | High SES | 0.004 | 0.009 | 0.67 |  |  |
| rs2176598 | Low SES | 0.016 | 0.007 | 0.018 | 0.30 | 0.33 |
|  | High SES | 0.028 | 0.007 | 2x10^-5^ |  |  |
| rs2207139 | Low SES | 0.037 | 0.008 | 2x10^-6^ | 0.38 | 0.41 |
|  | High SES | 0.042 | 0.008 | 5x10^-8^ |  |  |
| rs2245368 | Low SES | 0.028 | 0.008 | 3x10^-4^ | 0.57 | 0.60 |
|  | High SES | 0.016 | 0.008 | 0.046 |  |  |
| rs2287019 | Low SES | 0.028 | 0.008 | 2x10^-4^ | 0.29 | 0.32 |
|  | High SES | 0.041 | 0.008 | 5x10^-8^ |  |  |
| rs2365389 | Low SES | 0.035 | 0.006 | 2x10^-9^ | 0.94 | 0.95 |
|  | High SES | 0.024 | 0.006 | 4x10^-5^ |  |  |
| rs2650492 | Low SES | 0.020 | 0.006 | 0.002 | 0.97 | 0.97 |
|  | High SES | 0.019 | 0.006 | 0.004 |  |  |
| rs2820292 | Low SES | 0.021 | 0.006 | 4x10^-4^ | 0.73 | 0.75 |
|  | High SES | 0.019 | 0.006 | 0.001 |  |  |
| rs29941 | Low SES | 0.007 | 0.006 | 0.22 | 0.049 | 0.06 |
|  | High SES | 0.026 | 0.006 | 2x10^-5^ |  |  |
| rs3101336 | Low SES | 0.023 | 0.006 | 1x10^-4^ | 0.045 | 0.06 |
|  | High SES | 0.031 | 0.006 | 1x10^-7^ |  |  |
| rs3736485 | Low SES | 0.014 | 0.006 | 0.014 | 0.08 | 0.10 |
|  | High SES | 0.008 | 0.006 | 0.18 |  |  |
| rs3810291 | Low SES | 0.026 | 0.006 | 3x10^-5^ | 0.047 | 0.06 |
|  | High SES | 0.029 | 0.006 | 2x10^-6^ |  |  |
| rs3817334 | Low SES | 0.027 | 0.006 | 3x10^-6^ | 0.040 | 0.06 |
|  | High SES | 0.034 | 0.006 | 8x10^-9^ |  |  |
| rs3849570 | Low SES | 0.004 | 0.006 | 0.50 | 0.32 | 0.35 |
|  | High SES | 0.016 | 0.006 | 0.009 |  |  |
| rs4256980 | Low SES | 0.017 | 0.006 | 0.006 | 0.20 | 0.23 |
|  | High SES | 0.024 | 0.006 | 6x10^-5^ |  |  |
| rs4740619 | Low SES | 0.016 | 0.006 | 0.007 | 0.81 | 0.82 |
|  | High SES | 0.016 | 0.006 | 0.007 |  |  |
| rs543874 | Low SES | 0.040 | 0.007 | 2x10^-8^ | 0.008 | 0.013 |
|  | High SES | 0.056 | 0.007 | 4x10^-15^ |  |  |
| rs6477694 | Low SES | 0.000 | 0.006 | 0.99 | 0.042 | 0.06 |
|  | High SES | 0.014 | 0.006 | 0.023 |  |  |
| rs6567160 | Low SES | 0.046 | 0.007 | 2x10^-11^ | 0.003 | 0.005 |
|  | High SES | 0.060 | 0.007 | 1x10^-18^ |  |  |
| rs657452 | Low SES | 0.017 | 0.006 | 0.005 | 0.79 | 0.80 |
|  | High SES | 0.012 | 0.006 | 0.048 |  |  |
| rs6804842 | Low SES | 0.008 | 0.006 | 0.16 | 0.77 | 0.78 |
|  | High SES | 0.010 | 0.006 | 0.08 |  |  |
| rs7138803 | Low SES | 0.037 | 0.006 | 5x10^-10^ | 0.85 | 0.86 |
|  | High SES | 0.030 | 0.006 | 4x10^-7^ |  |  |
| rs7141420 | Low SES | 0.023 | 0.006 | 9x10^-5^ | 0.34 | 0.37 |
|  | High SES | 0.014 | 0.006 | 0.019 |  |  |
| rs7243357 | Low SES | 0.023 | 0.008 | 0.002 | 0.16 | 0.19 |
|  | High SES | 0.004 | 0.008 | 0.59 |  |  |
| rs758747 | Low SES | 0.007 | 0.007 | 0.27 | 0.62 | 0.64 |
|  | High SES | 0.021 | 0.007 | 0.001 |  |  |
| rs7599312 | Low SES | 0.015 | 0.007 | 0.025 | 0.49 | 0.52 |
|  | High SES | 0.024 | 0.007 | 3x10^-4^ |  |  |
| rs7899106 | Low SES | 0.033 | 0.013 | 0.014 | 0.87 | 0.88 |
|  | High SES | 0.015 | 0.013 | 0.27 |  |  |
| rs9400239 | Low SES | 0.015 | 0.006 | 0.020 | 0.51 | 0.53 |
|  | High SES | 0.019 | 0.006 | 0.003 |  |  |
| rs9581854 | Low SES | 0.012 | 0.007 | 0.10 | 0.08 | 0.10 |
|  | High SES | 0.016 | 0.008 | 0.029 |  |  |

**Supplementary table 9:** Differences in BMI by allele (inverse normalised scale) for TDI in the CoLaus Study, occupational status in the 1958 Birth Cohort and the UK Biobank and educational years in the UK Biobank

| **Study** | **Obesogenic category** | **N** | **BMI (SD)** | **Per-allele Beta** | **SE** | **P association** | **P interaction*** | **P Interaction Robust**** |
| --- | --- | --- | --- | --- | --- | --- | --- | --- |
| CoLaus | High SES based on TDI | 2,623 | 25.53  (4.33) | 0.030 | 0.004 | 6x10^-15^ | 0.35 | 0.34 |
|  | Low SES based on TDI | 2,614 | 26.18  (4.78) | 0.022 | 0.004 | 1x10^-8^ |  |  |
| UK Biobank | High job class | 38,942 | 27.15  (4.57) | 0.025 | 0.001 | <1x10^-15^ | 0.78 | 0.79 |
|  | Low job class | 37,374 | 27.68  (4.89) | 0.024 | 0.001 | <1x10^-15^ |  |  |
| 1958 Birth Cohort | High job class | 2,873 | 27.17  (4.55) | 0.026 | 0.003 | 2x10^-14^ | 0.62 | 0.62 |
|  | Low job class | 3,298 | 27.55  (5.10) | 0.024 | 0.003 | 1x10^-12^ |  |  |
| UK Biobank | High educational years (19-20) | 55,203 | 27.15  (4.67) | 0.024 | 0.001 | <1x10^-15^ | 0.76 | 0.76 |
|  | Low educational years (<=15) | 63,572 | 27.86  (4.93) | 0.023 | 0.001 | <1x10^-15^ |  |  |

BMI adjusted for age, sex, ancestral principal components and assessment centre location. Models additionally adjusted for genotyping platform

* Interaction p-value

** Interaction p-value accounting for heteroscedasticity using robust standard errors

**Supplementary table 10**: Change in BMI (single inverse normal scale) per allele increase in the BMI GRS when the obesogenic environments were dichotomised at approximately 25% low risk, 75% high risk.

| **Trait** | **Obesogenic category** | **N** | **Beta** | **SE** | **P association** | **P interaction*** |
| --- | --- | --- | --- | --- | --- | --- |
| Percentage fat | Low risk | 11592 | 0.024 | 0.002 | <1x10^-15^ | 0.93 |
|  | High risk | 34776 | 0.023 | 0.001 | <1x10^-15^ |  |
| Percentage protein | Low risk | 11591 | 0.022 | 0.002 | <1x10^-15^ | 0.68 |
|  | High risk | 34777 | 0.023 | 0.001 | <1x10^-15^ |  |
| Western diet | Low risk | 23510 | 0.021 | 0.001 | <1x10^-15^ | **0.029** |
|  | High risk | 70530 | 0.025 | 0.001 | <1x10^-15^ |  |
| IPAQ | Low risk | 27298 | 0.022 | 0.001 | <1x10^-15^ | 0.08 |
|  | High risk | 81844 | 0.023 | 0.001 | <1x10^-15^ |  |
| Sedentary time | Low risk | 30520 | 0.023 | 0.001 | <1x10^-15^ | 0.40 |
|  | High risk | 89168 | 0.024 | 0.001 | <1x10^-15^ |  |
| TV | Low risk | 53537 | 0.022 | 0.001 | <1x10^-15^ | **0.007** |
|  | High risk | 65299 | 0.025 | 0.001 | <1x10^-15^ |  |
| Vigorous activity | Low risk | 21676 | 0.021 | 0.001 | <1x10^-15^ | **0.003** |
|  | High risk | 87511 | 0.024 | 0.001 | <1x10^-15^ |  |
| TDI | Low risk | 29,946 | 0.022 | 0.001 | <1x10^-15^ | **4x10^-4^** |
|  | High risk | 89,787 | 0.024 | 0.001 | <1x10^-15^ |  |

* Interaction P-value calculated using the BMI GRS * dichotomous variable. Presented p-values were calculated with robust standard errors

**Supplementary table 11:** Change in BMI per allele increase in the BMI genetic risk score in individuals adjusted for smoking status (never, former and current) and the other obesogens that demonstrated significant interactions.

| **Trait** | **Obesogenic category** | **N** | **Beta (SE)** | **P association** | **P interaction robust**  **(P interaction robust*)** |
| --- | --- | --- | --- | --- | --- |
| IPAQ | >1845 MET minutes per week | 53,822 | 0.022 (0.001) | <1x10^-15^ | 2x10^-5^  (0.014) |
|  | <1845 MET minutes per week | 53,768 | 0.025 (0.001) | <1x10^-15^ |  |
| TV watching | <4 hours daily | 80,837 | 0.022 (0.001) | <1x10^-15^ | 0.026  (0.94) |
|  | >4 hours daily | 36,243 | 0.026 (0.001) | <1x10^-15^ |  |
| Composite score* | Low risk | 42,738 | 0.022 (0.001) | <1x10^-15^ | 1x10^-4^  (0.51) |
|  | High risk | 42,666 | 0.025 (0.001) | <1x10^-15^ |  |
| TDI | High SEP TDI<-2.294 | 59,872 | 0.022 (0.001) | <1x10^-15^ | 4x10^-7^  (3x10^-6^) |
|  | Low SEP TDI>-2.294 | 59,861 | 0.025 (0.001) | <1x10^-15^ |  |
| Sun protection use | Usually or always use | 68,507 | 0.022 (0.001) | <1x10^-15^ | 8x10^-4^ |
|  | Never or sometimes use | 50,561 | 0.025 (0.001) | <1x10^-15^ | (0.005) |

BMI adjusted for age, sex, ancestral principal components and assessment centre location. Models additionally adjusted for genotyping platform, smoking status and the obesogens demonstrating significant interaction with BMI genetics (e.g. for TDI it is adjusted for IPAQ, TV watching and the composite score). *P-value for interaction including adjustment for interaction terms for the other obesogens and smoking status.

| **Supplementary table 12:** Change in BMI (natural scale kg/m^2^) per allele increase in the BMI genetic risk score | | | | | | | | |
| --- | --- | --- | --- | --- | --- | --- | --- | --- |
| **Trait** | **Obesogenic category** | **N** | **Beta** | **SE** | **P association** | **P interaction** | **P Interaction Robust*** |  |
| Fizzy drink | None daily | 39975 | 0.11 | 0.004 | <1x10^-15^ | 0.28 | 0.34 |  |
|  | >1 glass daily | 6393 | 0.12 | 0.011 | <1x10^-15^ |  |  |  |
| Fried food consumption | None daily | 31821 | 0.11 | 0.005 | <1x10^-15^ | 0.63 | 0.65 |  |
|  | >1 meal daily | 14547 | 0.11 | 0.007 | <1x10^-15^ |  |  |  |
| Percentage fat^ | Low risk | 23194 | 0.11 | 0.005 | <1x10^-15^ | 0.33 | 0.36 |  |
|  | High risk | 23174 | 0.11 | 0.006 | <1x10^-15^ |  |  |  |
| Percentage protein^ | Low risk | 23188 | 0.10 | 0.006 | <1x10^-15^ | 0.50 | 0.55 |  |
|  | High risk | 23180 | 0.11 | 0.006 | <1x10^-15^ |  |  |  |
| Western diet^ | Low risk | 47027 | 0.11 | 0.004 | <1x10^-15^ | **0.006** | **0.008** |  |
|  | High risk | 47013 | 0.12 | 0.004 | <1x10^-15^ |  |  |  |
| IPAQ | >1845 MET minutes per week | 54573 | 0.09 | 0.003 | <1x10^-15^ | **5x10^-13^** | **9x10^-11^** |  |
|  | <1845 MET minutes per week | 54569 | 0.12 | 0.004 | <1x10^-15^ |  |  |  |
| Sedentary time | <5 hours daily | 63343 | 0.10 | 0.003 | <1x10^-15^ | **2x10^-6^** | **2x10^-5^** |  |
|  | >5 hours daily | 56345 | 0.12 | 0.004 | <1x10^-15^ |  |  |  |
| TV watching | <4 hours daily | 82022 | 0.10 | 0.003 | <1x10^-15^ | **3x10^-12^** | **8x10^-10^** |  |
|  | >4 hours daily | 36814 | 0.13 | 0.005 | <1x10^-15^ |  |  |  |
| Vigorous activity | >1 hour weekly | 35183 | 0.09 | 0.004 | <1x10^-15^ | 0.13 | 0.20 |  |
|  | <1 hour weekly | 74004 | 0.12 | 0.003 | <1x10^-15^ |  |  |  |
| Measured physical activity* | Low risk | 9,632 | 0.09 | 0.007 | <1x10^-15^ | **6x10^-6^** | **0.049** |  |
|  | High risk | 9,636 | 0.12 | 0.009 | <1x10^-15^ |  |  |  |
| Townsend Derivation Index | High SES TDI<-2.295 | 59,872 | 0.10 | 0.003 | <1x10^-15^ | **5x10^-17^** | **7x10^-14^** |  |
|  | Low SES TDI>-2.295 | 59,861 | 0.13 | 0.004 | <1x10^-15^ |  |  |  |
| Sun protection use | Usually or always use | 68,507 | 0.10 | 0.003 | <1x10^-15^ | **2x10^-4^** | **4x10^-4^** |  |
|  | Never or sometimes use | 50,561 | 0.12 | 0.004 | <1x10^-15^ |  |  |  |

Residuals were taken of BMI adjusted for age, sex, ancestry principal components and centre location. The models were further adjusted for genotyping platform at runtime.

*P-value for interaction term using robust standard errors in the regression model.

^ Data were split on the basis of an arbitrary median value

**References**

1 Bowden, J., Davey Smith, G. & Burgess, S. Mendelian randomization with invalid instruments: effect estimation and bias detection through Egger regression. *International journal of epidemiology* **44**, 512-525, doi:10.1093/ije/dyv080 (2015).

2 Locke, A. E. *et al.* Genetic studies of body mass index yield new insights for obesity biology. *Nature* **518**, 197-206, doi:10.1038/nature14177 (2015).
